# Impact of inflammatory preconditioning on murine microglial proteome response induced by focal ischemic brain injury

**DOI:** 10.3389/fimmu.2024.1227355

**Published:** 2024-04-09

**Authors:** Dario Lucas Helbing, Fabienne Haas, Emilio Cirri, Norman Rahnis, Therese Thuy Dung Dau, Erika Kelmer Sacramento, Nova Oraha, Leopold Böhm, Trim Lajqi, Pascal Fehringer, Helen Morrison, Reinhard Bauer

**Affiliations:** ^1^ Institute of Molecular Cell Biology, Jena University Hospital, Friedrich Schiller University, Jena, Germany; ^2^ Leibniz Institute on Aging, Fritz Lipmann Institute, Jena, Germany; ^3^ Department of Psychiatry and Psychotherapy, Jena University Hospital, Friedrich Schiller University Jena, Jena, Germany; ^4^ Center for Intervention and Research on Adaptive and Maladaptive Brain Circuits Underlying Mental Health (C-I-R-C), Jena-Magdeburg-Halle, Jena, Germany; ^5^ German Center for Mental Health (DZPG), Site Halle-Jena-Magdeburg, Jena, Germany; ^6^ Department of Microbiology and Hospital Hygiene, Bundeswehr Central Hospital Koblenz, Koblenz, Germany; ^7^ Department of Neonatology, Heidelberg University Children’s Hospital, Heidelberg, Germany; ^8^ Faculty of Biological Sciences, Friedrich-Schiller University, Jena, Germany

**Keywords:** mice, inflammatory preconditioning, neuroinflammation, ischemic stroke, microglia, type I interferons, phagocytosis

## Abstract

Preconditioning with lipopolysaccharide (LPS) induces neuroprotection against subsequent cerebral ischemic injury, mainly involving innate immune pathways. Microglia are resident immune cells of the central nervous system (CNS) that respond early to danger signals through memory-like differential reprogramming. However, the cell-specific molecular mechanisms underlying preconditioning are not fully understood. To elucidate the distinct molecular mechanisms of preconditioning on microglia, we compared these cell-specific proteomic profiles in response to LPS preconditioning and without preconditioning and subsequent transient focal brain ischemia and reperfusion, – using an established mouse model of transient focal brain ischemia and reperfusion. A proteomic workflow, based on isolated microglia obtained from mouse brains by cell sorting and coupled to mass spectrometry for identification and quantification, was applied. Our data confirm that LPS preconditioning induces marked neuroprotection, as indicated by a significant reduction in brain infarct volume. The established brain cell separation method was suitable for obtaining an enriched microglial cell fraction for valid proteomic analysis. The results show a significant impact of LPS preconditioning on microglial proteome patterns by type I interferons, presumably driven by the interferon cluster regulator proteins signal transducer and activator of transcription1/2 (STAT1/2).

## Introduction

Cerebral ischemic injury is the second leading cause of death and a major cause of long-term disability, with increasing incidence in the young and aging (1–3). Whilst therapy of acute ischemic stroke induced by large artery occlusion has evolved significantly in recent years, recognizing the value of thrombolysis and/or thrombectomy in appropriately selected patients (4, 5), the benefit of reperfusion therapies is incomplete in about half of said cohort (6). Furthermore, ischemia-reperfusion injury is often iatrogenically induced after life-saving endovascular or cardiac procedures (7, 8). To combat predictable ischemic injury, preactivation of transient endogenous protective mechanisms [“classical preconditioning”, reviewed in (9–11)] induced by stimulation with low doses of an otherwise deleterious insult, has been shown to be effective in reducing and even preventing sequelae of subsequent injurious ischemia (12, 13).

Preconditioning (PC) therefore represents a prophylactic intervention with various modalities – including non-injurious ischemia or hypoxia, hypothermia, pharmacological agents, low doses of endotoxin (LPS) – by inducing mainly inflammatory responses that can limit tissue damage of subsequent transient ischemia/reperfusion (I/R) (14, 15). Notably, PC induced by immune activators Toll-like receptor (TLR) ligands has shown remarkable efficacy in inducing ischemic tolerance: Systemic administration of several TLR ligands, including LPS, prior to focal cerebral ischemia, profoundly reduces ischemic injury in rodent stroke models (16–20). However, the complex molecular mechanisms underlying PC are not fully understood.

Given such limited therapeutic options and suboptimal tools for diagnosis and prognosis of ischemic stroke (IS), new strategies to increase brain cell survival after stroke are essential and best achieved by identifying better targets through deeper mechanistic understanding of IS pathophysiology (21, 22). High-throughput technologies, such as proteomics, have become crucial in unraveling key interactions between different molecular elements in complex biological contexts, such as IS and PC (23).

Previous studies suggest that PC effects of various origins mainly require innate immune pathways, including TLRs and type I interferons (IFN) (13, 24). Microglia, the CNS-resident neuro-immune cells that express these key innate immune receptors and modulators, respond quite early and extremely sensitively to immune-competent signals such as damage-associated molecular patterns (DAMPs) and pathogen-associated molecular patterns (PAMPs), through memory-like differential epigenetic and immunometabolic reprogramming (25–27). It has been shown that PC-dependent specific gene expression signatures in microglia prompt a functional shift of these mutant cells towards an immunomodulatory or protective phenotype (28). However, changes in messenger ribonucleic acid (mRNA) levels detected by transcriptomics may not correlate with similar changes in protein levels. As proteins perform most essential cellular functions, the composition of the proteome carries critical information about the state of an organism. The proteome is highly dynamic and adapts to changes in the microenvironment through transcriptional, translational, post-translational and degradational processes (22).

**Figure 1:**
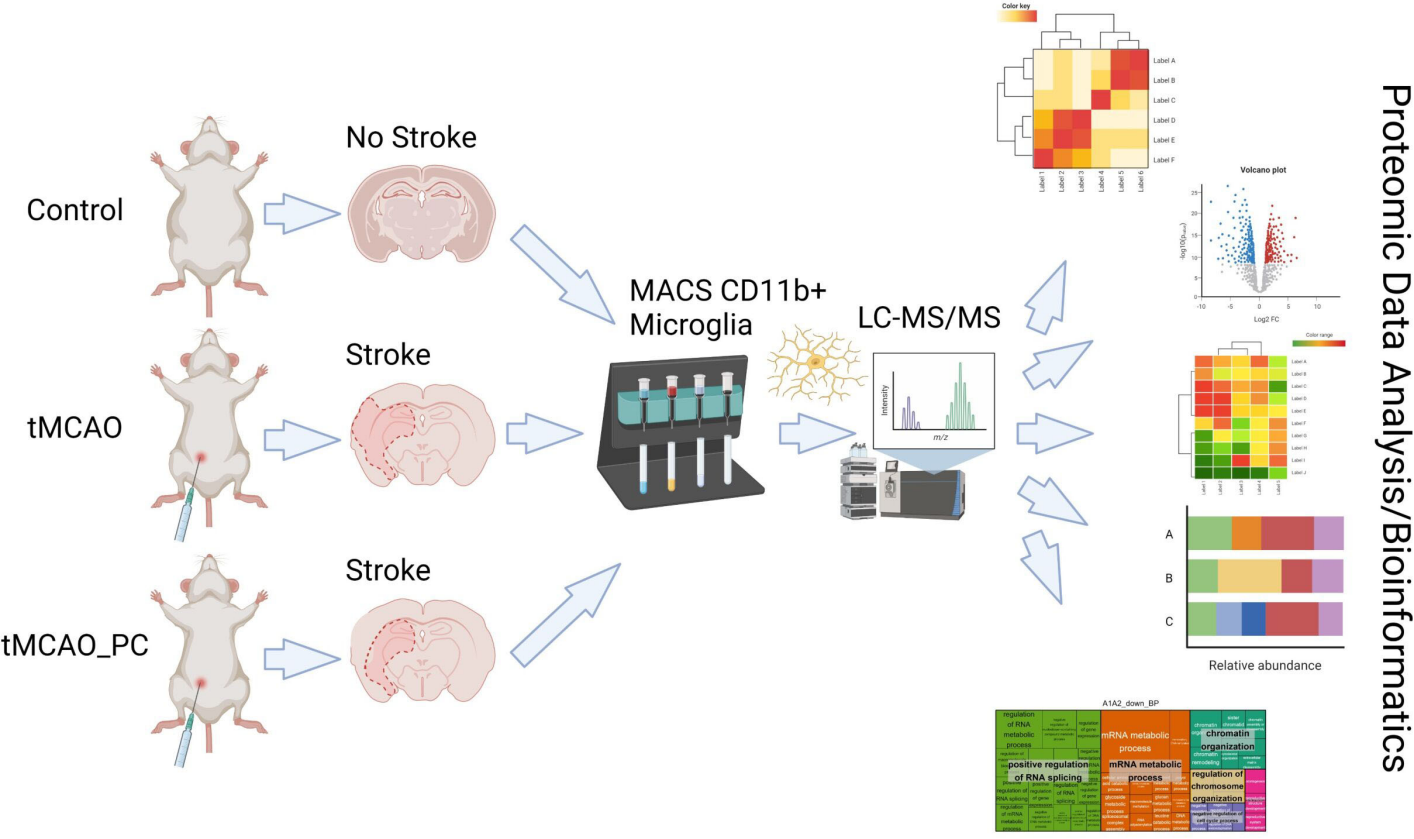
Schematic synopsis of performed experimental strategy to perform proteomic analysis of microglial response on temporal focal brain ischemia and reperfusion, without (tMCAO) or with previous LPS-induced inflammatory preconditioning (tMCAO_PC). (This figure was created under an institute license (Leibniz Institute on Aging Jena) with BioRender.com).

We focused on the impact of endogenous ischemic protection induced by LPS-induced PC, known to induce a multifaceted reprogramming of brain tissue at risk, leading to markedly reduced tissue destruction by subsequent IS. Thus, we applied a proteomic analysis based on isolated microglia obtained from mouse brains by cell sorting, coupled to mass spectrometry for identification and quantification. This approach was applied to a mouse model of focal brain ischemia and reperfusion by transient middle cerebral artery occlusion (tMCAO), without or after LPS-induced PC (tMCAO_PC), using various bioinformatic analyses to characterize microglial proteome remodeling in response to tMCAO and tMCAO_PC (**Figure 1**). This deductive approach was completed by targeted analyses of cell culture studies and of brain slice immunohistochemical (IHC) quantifications. This strategy enabled identification of specific effects of LPS-induced PC on transient brain I/R and potential neuroprotective effects mediated by microglial reprogramming.

Our data confirm that LPS-induced PC caused a significant reduction in infarct volume. The brain cell separation method used is suited to obtaining an enriched microglial cell fraction for valid proteomic analysis. The results show a significant impact of type I IFN on brain I/R-induced and LPS-induced PC modified microglial proteome patterns, presumably driven by cluster 3 regulators STAT1/2.

## Materials and methods

### Animals and experimental procedures

Male 12- to 16-week-old C57BL/6J mice were used in this study. The animal procedures were performed according to the guidelines of Directive 2010/63/EU of the European Parliament on the protection of animals used for scientific purposes. Experiments were approved by the Thuringian State Office for Food Safety and Consumer Protection. Efforts were made to reduce the number of animals used and their suffering. All surgeries were performed under appropriate anesthesia (see below).

Animals were introduced at least one week before commencing the interventions, to ensure appropriate acclimatization (29) and housed at neutral ambient temperature (30 ± 0.5°C) (30) during the entire experimental period.

Following acclimatization, mice were injected randomly with LPS (0.8 µg/g b.w., i.p.) for induction of immunological preconditioning (31), or physiological saline (NaCl) as a single intraperitoneal injection. Clinical status was assessed 48h, 24h and immediately before unilateral tMCAO according to (32, 33). Specifically, clinical status was assessed using a clinical severity score by scoring the status of spontaneous activity (Grade 1, No signs of illness, active, strong; Grade 2, Low-grad of illness, less active with occasional interruptions in activity; Grade 3, Mid-grade of illness, slow, sleepy, moves with difficulty; Grade 4, High-grade of illness, lethargic, motionless, no movement), reaction to exogenous stimuli (Grade 1, curious, quick movements; Grade 2, educed alertness, but adequate response; Grade 3, limited and delayed; Grade 4, none) and posture (Grade 1, normal; Grade 2, slightly hunched; Grade 3, hunched; Grade 4, severely hunched). Additionally, NaCl (500 μl) was injected subcutaneously immediately after LPS administration and after 24 h and 48 h. Meloxicam (Metacam®), used to prevent or treat pain, was administered orally 1-2mg/kg after LPS/saline injection, 24h and 48h.

### Focal cerebral ischemia/reperfusion

Three days later animals received transient focal brain ischemia and reperfusion by tMCAO. Prior to surgery, mice received meloxicam 1-2mg/kg orally. Subsequently, animals were anesthetized with 2.5% isoflurane for induction and 1.5% isoflurane for maintenance in 70/30% nitrous oxide/oxygen, administered by mask. Rectal temperature was maintained at 36.5 to 37°C with a feedback-controlled heating blanket. tMCAO was induced using the intraluminal filament technique (34). Briefly, the right common carotid artery, the external carotid artery and the internal carotid artery (ICA) were dissected from surrounding tissue. A 7–0 nylon monofilament (70SPRe, Doccol Corp, USA) was inserted into the ICA (11 mm) to occlude the middle cerebral artery. Operation time per animal did not exceed 15 min. The intraluminal suture was left *in situ* for 45 min. Animals were re-anesthetized and the occluding monofilament withdrawn to allow reperfusion. Animals were allowed to survive for 48 hours. Two hours post recovery from anesthesia, as well as 24 and 48 hours later, neurological deficits were scored in order to verify correct tMCAO induction by a modified Bederson score (35, 36) (scoring system: 0, no deficit; 1, forelimb flexion; 2, unidirectional circling; 3, longitudinal spinning; 4, no movement/death). Mice were excluded from analysis when subarachnoid hemorrhage was macroscopically observed during brain harvesting, or died before the end of the allotted reperfusion time. No difference was observed in exclusion rates between the groups. 48 hours after reperfusion, brains were harvested for subsequent immunohistochemical analyses or microglial cell sorting, accordingly.

### Histopathology and immunohistochemistry

#### Staining procedures

Mice were deeply anesthetized and perfused with 4% paraformaldehyde (PFA) in phosphate buffer, after rinsing with phosphate-buffered saline (PBS) by cardiac puncture via the left ventricle. Brains were removed immediately after fixation and postfixed for 5 h in 4% PFA at 4°C. After cryoprotection in PBS containing 30% sucrose, brains were frozen in methylbutane at −30°C and stored at −80°C. Whole brains were then cut by coronal sections at 40 μm on a freezing microtome (Microm International GmbH, Thermo Fisher Scientific, Germany). Free-floating sections were kept in 24-well plates in antifreeze-solution at -20°C until further use. For immunohistochemical stainings sections were treated with tris(hydroxymethyl)aminomethane buffered saline containing 10% normal donkey or goat serum, 1% bovine serum albumin, and 0.2% Triton X-100. Sections were incubated with the desired primary antibody at 4°C overnight, followed with the associated secondary antibody at 4°C for 1 h, and visualized by fluorescence imaging. Control sections were incubated with the blocking solution in the absence of the respective primary antibody. The following primary antibodies were used: mouse anti- microtubule-associated protein 2 (MAP2) (1:1000) antibody (Sigma-Aldrich Chemie Gmbh Munich, Germany) for MAP2 staining, goat polyclonal anti-ionized calcium-binding adaptor molecule 1 (Iba1)Iba-1 (1:750) antibody (Abcam, Cambridge, UK) for Iba1 staining, rabbit monoclonal anti-STAT1 (1:200) antibody (Cell Signaling Technology, Danvers, USA) for STAT1 staining and Hoechst 33342 (Fisher Scientific GmbH, Schwerte, Germany) for nucleic acid staining (NAS). For visualization, the Biotin-SP AffiniPure Donkey Anti-Mouse secondary antibody for MAP2 and the secondary fluorescent isotype-specific antibodies Alexa Fluor™ 488 for Iba1 and Alexa Fluor™ 546 for STAT1 were used.

#### Infarct volume estimation

The MAP2 immunostained slices were displayed with a Olympus CX41 Upright Microscope and mounted digital Olympus DP50 camera (Evident Europe GmbH, Hamburg, Germany). Planimetric measurements (ImageJ software, National Institutes of Health, Bethesda, MD) blinded to the treatment groups were used to calculate lesion volumes, which were then corrected for brain edema.

#### Microglial fluorescent intensity measurements

Triple immunofluorescence stainings for Iba1, STAT1 and NAS in all brain sections under consideration were simultaneously performed. The stained brain sections were completely scanned one after the other and with identical settings using Axio Scan.Z1 digital slide scanner (Carl Zeiss Microscopy, Jena, Germany) and Zeiss Zen 2.3 Slidescan software. The images were created using a Plan-Apochromat 20x/0.8 objective with a final resolution of 0.23 µm per pixel. Measurements of microglial fluorescence intensities were carried out on the brain slices with the greatest extent of the ischemic infarction. The respective area of ischemic brain damage was determined using the adjacent MAP2 staining section. NAS channel was set as a focus and stitching reference to acquire whole section high-resolution images. Z-stacks were automatically merged via maximum intensity projection and viewed by Zeiss Zen 2.6 (blue edition) to extract a region of interest (ROI). Subsequently, images were analyzed with Fiji (37). Measurements were conducted semi-automated with custom-made macros. In brief, after background subtraction, binary masks from Iba1+ cells were generated and placed as overlay on the STAT1 channel to analyze the fluorescence intensity of the corresponding grayscaled channels. Measurements of microglial fluorescence intensities for STAT1 were therefor performed within the infarction core using the Iba1-positive cells within the respective ROI. This was done by creating a template of the Iba1-positive cells within the ROI, which acted as a measurement scheme to determine STAT1 fluorescence in Iba1-positive cells. Fluorescence intensity was normalized to the averaged signal intensities obtained from respective brain regions of untreated control animals.

### Magnetic-activated cell sorting of primary microglial cells

For microglial cell isolation mice brain vessels were rinsed transcardially by ice-cold PBS perfusion and brains harvested immediately after. The cerebellum and meninges were removed and split in the median sagittal plane. In each case, right hemisphere containing infarction, when mice suffered from tMCAO or being unaffected (sham cohort), was sliced into small pieces and transferred to customer-specified tubes for magnetic-activated cell sorting (MACS) (Adult Brain Dissociation Kit #130-107-677 and Cd11b MicroBeads, mouse #130-093-634, Miltenyi Biotec, Bergisch Gladbach Germany). Samples were processed per manufacturer’s instructions with the following modifications: Initially 1% HEPES 1M was added for pH buffering to the D-PBS and PB buffer. Samples were transferred to a C Tube containing the enzyme mix I (enzyme P and buffer Z from the Adult Brain Dissociation Kit #130-107-677, Miltenyi Biotec, mixed according to manufacturer’s instructions). Immediately thereafter, enzyme mix II (enzyme A and buffer Y, from the Adult Brain Dissociation Kit #130-107-677, Miltenyi Biotec, mixed according to manufacturer’s instructions) was added and the samples processed using a gentleMACS Octo Dissociator with Heaters (gentleMACS Program 37C_ABDK_01). The samples were temporarily centrifuged, then triturated 10 times with two different-lumen Pasteur pipettes. Next, the cell suspensions were filtrated with a MACS SmartStrainer and 10 ml of cold D-PBS added, followed by centrifugation for 10 min at 300 g, 4°C. The supernatant was removed and the cell pellets resuspended in 3100 µl D-PBS. Subsequently, 900 µl of Debris Removal Solution was added and mixed. The mixture was gently overlayed with 4 ml of D-PBS and mixing of the phases was avoided. Three phases formed after a centrifugation step at 3000 g for 10 min, 4°C. The upper two phases were removed and 15 ml of D-PBS was added to the remaining cell pellet, followed by three inversions of the tube. Following another centrifugation step (1000 g for 10 min, 4°C), the cell pellet was resuspended in 90 µl PB buffer. 10 µl of CD11b (Microglia) MicroBeads were added and mixed. This mixture was incubated for 15 min at 4°C in the dark and mixed every 5 min by slightly flicking the tube. The cells were washed by adding 1 ml of PB buffer, centrifuged at 300 g for 5 min, 4°C, then resuspended in 500 µl of PB buffer.

For flow cytometric analysis, 2x 20 µl samples were collected (unstained original fraction and original fraction (“OF”)). Next, MS Columns were placed onto a MACS^®^ Separator, and prepared by rinsing with 500 µl of PB buffer. The whole brain cell suspension was then applied to the columns and the flow-through containing unlabeled cells collected, hereafter referred to as non-target cell fraction (“NTCF”). 100 µl of cell suspension was collected from this fraction for FACS analysis. The column was washed 3x with 500 µl of PB buffer, removed from the separator and placed into a collection tube. 1 ml of D-PBS was pipetted onto the column and immediately afterwards the magnetically labeled CD11b positive cells were flushed out by pushing the plunger into the column. This step was repeated once. 100 µl cell suspension was collected from this fraction again for FACS analysis, hereafter referred to as “Cd11b+” ≙ CD11b positive fraction. This fraction was centrifuged at 300 g for 5 min, 4°C, followed by aspiration of the supernatant. The resulting cell pellet was snap-frozen in liquid nitrogen and stored at -80°C, pending further analysis.

### Flow cytometry and data analysis

Three different cell suspensions were obtained: (i) the original fraction of unstained cells before column separation, (ii) the non-target cell fraction consisting of the flow-through of CD11b negative cells and (iii) the CD11b positive fraction containing microglia. 100 µl of each cell suspension were used (original fraction contains 20 µl of total brain cell suspension with 80 µl of PB buffer added) for analysis of MACS-sorted primary microglial cells and 5 µl of CD11b+-APC antibody added (1:20). These samples were incubated for 10 min in a refrigerator, protected from light. The cell suspensions were then washed by adding 1 ml of PB buffer, followed by centrifugation at 300 g for 5 min, 4°C. Next, the supernatant was completely aspirated and the cell pellet resuspended in 200 µl of PB buffer. Finally, the cells were analyzed by fluorescence measurement (BD FACSCantoTM System). The collected data were exported for further analysis and generation of scatter plots with FlowJo (version 10.2, BD Bioscience).

### Mass spectrometry

#### Sample preparation for proteomics

Snap frozen cell pellets were resuspended in lysis buffer (final concentration: 0.1 M HEPES/pH 8; 2% SDS; 0.1 M DTT) and vortexed. All samples were sonicated using a Bioruptor (Diagenode) (10 cycles with 1min on and 30s off with high intensity @ 20°C). For reduction and full denaturation of the proteins, the lysates were incubated at 95°C for 10 min, before treatment with iodacetamide (room temperature, in the dark, 30 minutes, 20 mM).Each sample was subsequently treated with 8 volumes of ice-cold acetone to 1 volume sample and left overnight at -20°C to precipitate the proteins. Next, samples were centrifuged at 14,000 rpm for 30 minutes, at 4°C. Following removal of the supernatant, the precipitates were washed twice with 200 μL of 80% acetone (ice cold). After each wash the samples were vortexed and centrifuged for 2 minutes at 4°C. The pellets were allowed to air-dry before being dissolved in digestion buffer (1M Guanidine, 100 mM HEPES, pH 8) with sonication (3 cycles in the Bioruptor as above) and incubated for 4 h with LysC (1:100 enzyme: protein ratio) at 37°C, with shaking at 600 rpm. Next, the samples were diluted 1:1 with deionized water (from a Milli-Q^®^ system) and incubated with trypsin (1:100 enzyme: protein ratio) for 16 h at 37°C. In the presence of a slow vacuum, the digests were acidified with 10% trifluoroacetic acid and desalted (Waters Oasis^®^ HLB μElution Plate 30μm). The columns were conditioned with 3x100 μL solvent B (80% acetonitrile; 0.05% formic acid) and equilibrated with 3x 100 μL solvent A (0.05% formic acid in milliQ water), before the samples were loaded, washed 3 times with 100 μL solvent A then eluted into PCR tubes with 50 μL solvent B. These eluates were dried down with a speed vacuum centrifuge and dissolved in 50 μL 5% acetonitrile, 95% milliQ water, with 0.1% formic acid, prior to analysis by LCMS/MS.

#### LC-MS data dependent and independent acquisition

For data dependent acquisition (DDA), peptides were separated using UltiMate 3000 UPLC system (Thermo Fisher Scientific), fitted with a trapping column (Waters nanoEase M/Z Symmetry C18, 5μm, 180 μm x 20 mm) and an analytical column (Waters nanoEase M/Z Peptide C18, 1.7μm, 75μm x 250mm). Solvent A consisted of water, 0.1% formic acid, while solvent B consisted of 80% (v/v) acetonitrile, 0.08% formic acid. The samples (500 ng) were loaded with a constant flow of solvent A at 5 μL/min onto the trapping column, with a trapping time of 6 minutes. Peptides were eluted via the analytical column at a constant flow of 0.3 μL/min. During this step, the percentage of solvent B increased in a linear fashion from 1% to 7% within 7 minutes, before further increasing to 32% within 24 more minutes and finally to 50% within a further 8 minutes. The outlet of the analytical column was coupled directly to a Q exactive HF (Thermo Fisher Scientific) using the Proxeon nanospray source. The peptides were introduced into the mass spectrometer via a Pico-Tip Emitter 360 μm OD x 20 μm ID; 10 μm tip (New Objective) and a spray voltage of 2.2 kV applied. The capillary temperature was set at 300°C and the S-lens RF value to 60%. Full scan MS spectra with mass range 350-1650m/z were acquired in the Orbitrap at a resolution of 60,000 FWHM (= full width half maximum). The filling time was set at maximum of 20 ms with an automatic gain control (AGC) target value of 3x106 ions. A Top15 method (selecting the top 15 most intense m/z features) was applied to select precursor ions from the full scan MS for fragmentation (minimum AGC target of 1x 103 ions, normalized collision energy of 31%), quadrupole isolation (1.6 m/z) and measurement in the Orbitrap (resolution 15,000 FWHM, fixed first mass 120 m/z). Fragmentation was performed after accumulation of 2x105 ions or after filling time of 25 ms for each precursor ion (whichever occurred first). Only multiple charged (2+ -7+) precursor ions were selected for MS/MS. Dynamic exclusion was employed with maximum retention period of 30 s. Isotopes were excluded.

For data independent acquisition (DIA), 1 μg of reconstituted peptides were separated using a nanoAcquity UPLC (Waters, Milford, MA), which was coupled online to the MS. Peptide mixtures were separated in trap/elute mode, using a trapping (nanoAcquity Symmetry C18, 5 μm, 180 μm × 20 mm) and an analytical column (nanoAcquity BEH C18, 1.7 μm, 75 μm x 250 mm). The outlet of the latter was coupled directly to an Orbitrap Fusion Lumos mass spectrometer (Thermo Fisher Scientific, San Jose, CA) using the Proxeon nanospray source. Solvent A consisted of water, 0.1% formic acid and solvent B consisted of acetonitrile, 0.1% formic acid. The samples were loaded with a constant flow of solvent A, at 5 μL/min onto the trapping column with a trapping time of 6 min. Peptides were eluted via the analytical column at a constant flow of 300 nL/min. During this step, the percentage of solvent B increased in a nonlinear fashion from 0% to 40% within 120 min. Total runtime was 145 min, including cleanup and column re-equilibration. The peptides were introduced into the mass spectrometer via a Pico-Tip Emitter 360 µm OD x 20 µm ID; 10 µm tip (New Objective) and a spray voltage of 2.2 kV applied. The capillary temperature was set at 300°C and the RF lens set to 30%. Full scan MS spectra with mass range 350-1650 m/z were acquired in profile mode in the Orbitrap at a resolution of 120,000 FWHM. The filling time was set at maximum of 20 ms with an AGC target of 5 x 10^5^ ions. DIA scans were acquired with 40 mass window segments of differing widths across the MS1 mass range and the HCD collision energy set to 30%. MS/MS scan resolution in the Orbitrap was set to 30,000 FWHM with a fixed first mass of 200m/z after accumulation of 1x 106 ions, or after filling time of 70 ms (whichever occurred first). Data were acquired in profile mode. Tune version 2.1 and Xcalibur 4.1 were used for data acquisition and processing. The MS/MS scan resolution in the Orbitrap was set to 30k with an AGC target of 1 × 10^6^ and max injection time of 70 ms. All data acquisition was performed using XCalibur 4.0/Tune 2.1 (Thermo Fisher Scientific).

#### Mass spectrometry data analysis for DDA DIA samples

For DDA, data were analysed using MaxQuant (version 1.5.3.28) (38). MS/MS spectra were searched against the Mouse Swiss-Prot entries of the Uniprot KB (database release 2016_01, 16,756 entries), using the Andromeda search engine (39). A list of common contaminants was appended to the database search and the search criteria set as follows: full tryptic specificity was required (cleavage after lysine or arginine residues, unless followed by proline); 2 missed cleavages were allowed; oxidation (M) and acetylation (protein N-term) were applied as variable modifications, with mass tolerances of 20 ppm set for precursor and 0.5 Da for fragments. The reversed sequences of the target database were used as decoy database. Peptide and protein hits were filtered at a false discovery rate of 1% using a target-decoy strategy (40). The protein group intensities per protein (from the proteinGroups.txt output of MaxQuant) were used for further analysis and visualization, both were performed with R (41).

For DIA, acquired data were processed using Spectronaut Professional v13.10 (Biognosys AG). Raw files were searched using directDIA with Pulsar (Biognosys AG) against the mouse UniProt database (Mus musculus, entry only, release 2016_01), with a list of common contaminants appended, using default settings. Default Biognosys (BGS) factory settings were used for library generation. DIA data were searched using BGS factory settings, except: Proteotypicity Filter = Only Protein Group Specific; Major Group Quantity = Median peptide quantity; Major Group Top N = OFF; Minor Group Quantity = Median precursor quantity; Minor Group Top N = OFF; Data Filtering = Qvalue sparse; Normalization Strategy = Local normalization; Row Selection = Automatic. The candidates and protein report tables were exported from Spectronaut and used for further analysis as described below.

### Exploratory/descriptive data analysis

All data analysis steps were completed with R (41), unless otherwise stated. A PCA analysis and analysis of sample correlation was performed with the pcromp and cor function, respectively, to visualize the high dimensional data. The relevant comparisons were divided and specific abbreviations were assigned to each: A1 Wildtype stroke with NaCl treatment/Wildtype control; A2 Wildtype stroke with LPS priming/Wildtype control. Proteins were considered differentially expressed (“DEP”) when they were up- or down-regulated by a factor of 1.5 to the respective control value. This symmetrical definition of DEPs was ensured by transforming the fold changes to logarithmic values with a base of 2 (42), resulting in a cut-off of │log2 FC│≥ 0.58. Furthermore, proteins with a q-value of ≤ 0.05 were considered as DEPs. Heatmaps were generated with the ComplexHeatmap package (43) in order to visualize different patterns and shared regulation of gene clusters. The protein quantities were z-score normalized for all samples by protein row-wise. The dendrogram was reordered with the R package dendsort (44) for appropriate visualization. Correlation analysis of all and significantly changed proteins was achieved using the cor.test function from the R stats package and visualized by a customized function using the ggplot2 package. The correlation analysis was performed with the Spearman correlation method, since the Log2 fold changes of both datasets were not normally distributed. The intersections, as well as the uniquely changed DEPs, were retrieved by joining the A1 and A2 DEP lists with different functions from the dplyr package within the tidyverse package. These partitions were visualized with Venn diagrams using the draw.pairwise.venn function of the VennDiagram package (45), before being subjected to a gene ontology and KEGG pathway enrichment analysis.

### Proteomic assessment of microglia enrichment

The proteomic data harvested from the CD11b+ and the mixed cell suspension containing all other brain cells (NTCF), was compared to a high-resolution proteomics dataset of oligodendrocytes, astrocytes, cortical neurons and microglia (46). The cell-type specific proteome dataset was sorted for every protein to obtain the cell types showing the highest expression of the respective protein. Subsequently, the cell types showing the second highest expression were identified and a ratio for the respective protein between the expression values for these two cell types calculated. A protein was defined to be a microglia specific marker if this ratio showed at least a 5-fold higher expression in microglia than in the cell type with the second highest expression. This criterion was also applied to identify oligodendrocyte, astrocyte and cortical neuron specific marker proteins. The thus identified proteins of all other cell types were bound together for heatmap generation as “non-microglia proteins”. The obtained protein lists of cell-type specific marker proteins were then joined with the proteome of the CD11b+ samples and the mixed cell suspension to identify and count the number of cell-type specific proteins in the two different MACS fractions.

### Functional protein annotation and enrichment analyses

A gene enrichment and functional annotation analysis was performed by the DAVID platform (47) with a conversion success rate of >99,9%. The gene lists, obtained by DEP analysis as well as Venn analysis, were submitted to DAVID and are illustrated in **Table 1**. The corresponding datasets of all proteins from the A1 and A2 analyses – or the intersections of all in both comparisons – detected proteins were used instead of the total mus muscuclus genome as background dataset for gene ontology enrichment analysis, in order to obtain specifically the enriched gene ontology terms within the determined microglia proteome. GO-terms that describe biological processes, GO-terms that describe cellular components and KEGG pathways were integrated into further analysis. The analysis was restricted to level 5 gene ontology (GO) terms representing a trade-off between high coverage of differentially expressed genes per GO-term and likewise a specific description of a biological process. A default threshold of at least 2 counts per GO-term was used. Lists of Gene Ontology terms of the biological processes with a Fisher’s Exact p-value ≤ 0.1 were either subjected to REVIGO analysis (48) for semantic similarity analysis and reduction, or analyzed using the simplifyEnrichment R package as described by the developers (49). REVIGO analysis was achieved under the following parameters: medium list size (0.7) and mus musculus UniProt database as identifier with the by default preselected simRel score (50) used as semantic similarity measure. The resulting R scripts were modified to suit the way in which the graphical parameters should be displayed. Lists of enriched KEGG-pathways were filtered for a Fisher’s Exact p-value ≤ 0.05 and imported into R. A bubble plot was generated for visualization with a custom function using the ggplot2 package. Interesting enriched KEGG pathways were further visualized with the R package Pathview (51): Datasets were filtered for a q-value of ≤ 0.05 and relevant columns (Log2 Fold changes) of A1 and A2 were subsetted. The KEGG pathview figure of the respective pathway was downloaded and the proteomics data annotated via the gene symbols. The gene list of the gene ontology term “GO:0045335 phagocytic vesicle” was retrieved using the R package “org.Mm.eg.db” with a taxon filter for mus musculus.

**Table 1:** Protein lists for DAVID enrichment analysis.

	Up in A1	Up in A2	Down in A1	Down in A2
A1_up	yes			
A2_up		yes		
A1_down			yes	
A2_down				yes
A1A2upall	yes	yes	no	no
A1A2downall	no	no	yes	yes
A1_up_unique	yes	no	no	
A2_up_unique	no	yes		no
A1_down_unique	no		yes	no
A2_down_unique		no	no	yes

“yes” means that a protein from lists shown in the rows is also present in the list of the respective column; “no” means this is not the case and an empty box denotes no information can be stated as to whether or not a protein is present in the list of the respective column.

### Integration of other datasets for bioinformatic analyses

A dataset to determine the functional activation of microglia towards a pro- or anti-inflammatory state was retrieved, in which proteomic signatures of proinflammatory “classically activated” microglia and anti-inflammatory/”alternatively activated” microglia were determined (52). These gene lists were annotated to the list of proteins that were significantly regulated in either the A1 or A2 comparison via the UniProtIDs, to extract pro- and anti-inflammatory DEPs. The protein intensities of these proteins were z-score normalized, visualized with a heatmap and an analysis of differential protein abundance between protein sets belonging to the respective gene sets was conducted. Furthermore, a dataset of transcriptome profiles of isolated microglia exposed *in vivo* to Interferon-β was retrieved (53) and processed as described by the authors of the respective paper. The resultant list of 255 induced/upregulated genes in microglia by Interferon-β was provided a gene set for estimating the induction of a type I IFN responsive microglial state after tMCAO. Gene set enrichment analysis was performed using the fgsea R package (54) and visualization p using the gggsea package (55). Bar plots and statistical testing were completed as described above and in the respective figure legends. The final task involved performing a cluster specific analysis of the type I IFN network as determined previously (56). This dataset was obtained from the supplementary data (53) to extract cluster wise the assigned regulator and target proteins. These gene sets were matched to all proteins from the A1 or A2 comparison. Subsequently, the joined datasets were filtered for a q-value of < 0.05 for network visualization. Differential protein abundances of cluster 3 regulator and target proteins between the different experimental groups were estimated via statistical verification on the paired datasets.

### *In vitro* analyses

#### BV-2 cell culture, LPS stimulation

BV-2 cells, immortalized murine neonatal microglial cells, were cultured at 37°C and 5% CO_2_ in Dulbecco’s Modified Eagle’s Medium (DMEM) - high glucose from Sigma-Aldrich (#D6429, St. Louis, MO, USA) supplemented with 10% heat-inactivated fetal bovine serum (FBS, #PB-FCS-EU-0500, PeloBiotech; endotoxin-free and sterile-filtered), 1% penicillin/streptomycin, and 1% amphotericin B. After becoming adherent, cells were starved for 4h in DMEM medium without FCS. After starvation, the medium was changed to DMEM - high glucose from Sigma-Aldrich (#D6429, St. Louis, MO, USA). Thereafter, cells were stimulated with LPS for 3 hours and then lysed for RNA isolation as described below. A concentration of 100 ng/mL LPS (E. coli serotype 055:B5; #tlrl-pb5lps, InvivoGen, Toulouse, France) was used as optimal dose with highest viability for the stimulation of BV-2 cells (57).

#### Oxygen-glucose deprivation

OGD was induced as described previously (36, 58). Briefly, BV-2 cells were seeded in 6 well/plates (100.000 cells/well) and incubated over night at 37°C and 5% CO_2_ in DMEM high glucose supplemented with 10% FCS, 1% penicillin/streptomycin, and 1% amphotericin B. After becoming adherent, cells were starved for 4h in DMEM medium without FCS. After starvation, the medium was changed to DMEM without glucose (#11966025, Thermo Fischer Scientific, Waltham, MA, USA), and cells were incubated under hypoxic conditions at <0,1% O_2_ for 3h. After OGD, the medium was replaced by DMEM - high glucose, 1% penicillin/streptomycin, and 1% amphotericin B, and cells were incubated at normoxic conditions at 37°C and 5% CO_2_ for 3h (reoxygenation) without or with LPS. Thereafter, OGD- and LPS+OGD-treated cells were lysed for RNA isolation as described below.

#### RNA isolation and real-time qPCR

To determine gene expression levels, the total RNA was extracted using TRIzol Reagent (#15596018, Invitrogen, Carlsbad, CA, USA) following the manufacturer instructions as described (59, 60). RNA concentration and quality were checked using the LB916 Colibri+ Microvolume Spectrometer ver. 25B05 (Berthold Technologies, Bad Wildbad, Germany). During the whole procedure, RNase Away (#7003, Molecular BioProducts, San Diego, CA, USA) solution was used to flush pipettes and other equipment in order to prevent any contamination with other RNases or DNAs. Complementary DNA (cDNA) was synthesized using High-Capacity cDNA Reverse Transcription kits (#4368814, Applied Biosystems, Waltham, MA, USA). Real-time qPCR reaction was performed by using StepOnePlusTM real-time PCR System (Applied Biosystems, Waltham, MA, USA). The following primer pairs were used for this study, RN18S forward: CCAGAGCGAAAGCATTTGCC and RN18S reverse: GCATTGCCAGTCGGCATCGT, CyPD forward: GCAAGGATGGCAAGGATTGA and CyPD reverse: AGCAATTCTGCCTGGATAGC, STAT1 forward: TCACAGTGGTTCGAGCTTCAG and STAT1 reverse: GCAAACGAGACATCATAGGCA, STAT2 forward: TCCTGCCAATGGACGTTCG and STAT2 reverse: GTCCCACTGGTTCAGTTGGT, IL-1β forward: GGCAGGCAGTATCACTCATT and IL-1 β reverse: AAGGTGCTCATGTCCTCAT, IL-10 forward: ACCAGCTGGACAACATACTGC and IL-10 reverse: TCACTCTTCACCTGCTCCACT, IFN-α forward: TGACCTCAAAGCCTGTGTGATG and IFN-α reverse: AAGTATTTCCTCACAGCCAGCAG, IFN-β forward: CAGCTCCAAGAAAGGACGAAC and IFN-β reverse: GGCAGTGTAACTCTTCTGCAT. RN18S and CyPD were used as housekeeping genes. Relative gene expression was calculated using the comparative CT (2−ΔΔCT) method (61).

### Statistics

Data were presented as scatter dot plots, mean ± SEM, if not otherwise indicated. Shapiro-Wilk normality test was conducted before further statistical analysis was performed. Parametric (one-way ANOVA, Kruskal-Wallis ANOVA on Ranks, two-way ANOVA and repeated measures ANOVA with *Post-hoc* Holm-Sidak test, Tukey test, Student-Newman-Keuls test, unpaired Student’s t-test with Welch’s correction) or non-parametric tests (Friedman-test with *post-hoc* Wilcoxon signed-rank test and adjustment of p-values for multiple testing with the Bonferroni-Holm correction) were completed where appropriate. Statistical tests for proteomic analysis and functional annotation and enrichment analyses were performed as indicated above. Differences were considered significant when p < 0.05. The statistical analysis was achieved using Graphpad Prism 8.4 or R (41).

## Results

### LPS preconditioning reduced cerebral infarct size following I/R

**Figure 2:**
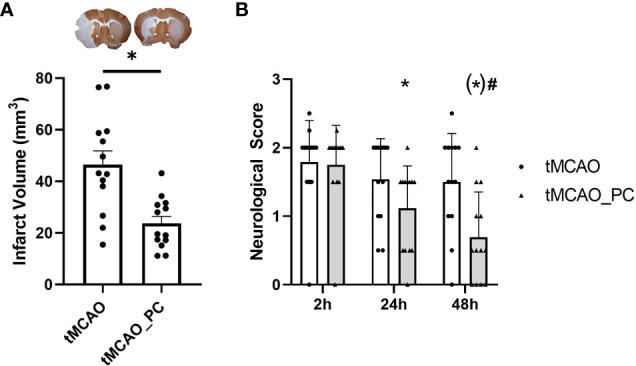
Reduction in infarct size and improved neurological recovery in LPS-preconditioned animals and tMCAO. **(A)** Reduced infarct volume in animals with LPS-induced preconditioning (n = 13 animals per condition, scatterplot, mean + SEM, statistical power: 0.99, effect size (Cohen’s d): 1.61, Unpaired Students t-test with Welch’s correction with * = p < 0.05, specifically p = 0.0012; upper panel: representative pictures of brain slices stained with MAP2 to indicate infarct size (pale region at the left side, respectively). **(B)** Time course of neurological deficits after tMCAO as detected by a modified Bederson score (35, 36) (n = 13 animals per condition, two-way ANOVA with p = 0.0002 for the factor time post-tMCAO and p = 0.025 for the factor preconditioning. *Post-hoc* Holm-Sidak test with * p < 0.05 for the comparison to 2h time point within each conditioning group (tMCAO or tMCAO_PC), p = 0.0084 for the comparison 2h vs. 24h tMCAO_PC, p < 0.0001 for the comparison 2h vs. 48h tMCAO_PC, p = 0.0508 for the comparison 48h vs. 24h in the tMCAO_PC group, # p < 0.05 for comparisons tMCAO vs. tMCAO_PC within each time point, (*) p = 0.0050 for the 48h time point.).

Two mouse cohorts received a 45 minutes lasting unilateral middle cerebral artery occlusion three days after either LPS-induced preconditioning (LPS-PC) or NaCl injection. Mice survived for 48 hours after tMCAO. Clinical status was assessed by scoring spontaneous activity, response to exogenous stimuli, and posture to assess the severity of LPS-induced systemic inflammation in a reproducible manner (32). LPS-PC only slightly worsened the clinical course prior to tMCAO (**Supplementary Table 1**), but led to an approximate halving of the infarct volume (**Figure 2A**). In addition, preconditioned animals displayed significantly better neurological recovery within the first 48h after tMCAO, as determined by modified Bederson score (35, 36) (**Figure 2B**). Therefore, LPS-PC caused the expected protection of ischemic brain injury.

### MACS-based microglia isolation

It is known that microglia play an essential role in mediating neuroprotective effects of various forms of preconditioning (25). However, the molecular mechanisms have not yet been sufficiently elucidated. We chose the deductive approach of microglial proteome analysis to better understand the complex intracellular processes resulting from LPS-PC and focal brain ischemia. To ensure high-quality proteomic profiles of isolated microglia with response to focal brain I/R and preconditioning, the magnetic-activated cell sorting-based isolation protocol for adult microglia was first validated by flow cytometric data analysis (**Supplementary Figures 1A–C**) and proteomic analyses (**Supplementary Figures 1D–G**) of microglia cells (CD11b+), as well as the CD11b negative cell fraction [CD11b-/NTCF (= non-target cell fraction)] comprised of astrocytes, oligodendrocytes and neurons. Principal component analysis of the proteomic profiles of both fractions revealed marked differences, proving the presence of different cell types (**Supplementary Figure 1D**). Integration of the proteome profiles determined here with previously determined proteome profiles of different cell types from adult mouse brain (46), revealed a distinct presence of microglia specific marker proteins in the CD11b+ fraction; non-microglia proteins were almost completely absent, whereas non-microglia proteins were mainly found in the CD11b- fraction (NTCF) (**Supplementary Figures 1E, G**). The same pattern was observed by investigating commonly used marker proteins for microglia (Aif1/Iba1), astrocytes (Gfap), oligodendrocytes (Cnp) and neurons (Tuj1/Tubb3) (**Supplementary Figure 1F**) – indicating a comparatively good enrichment of microglia by MACS-based separation and sparse contamination of non-microglial cells. Therefore, the isolation method used is suitable for obtaining an enriched microglia cell fraction for valid proteomics experiments.

### Differential proteomic response in microglia after tMCAO with or without LPS preconditioning

**Figure 3:**
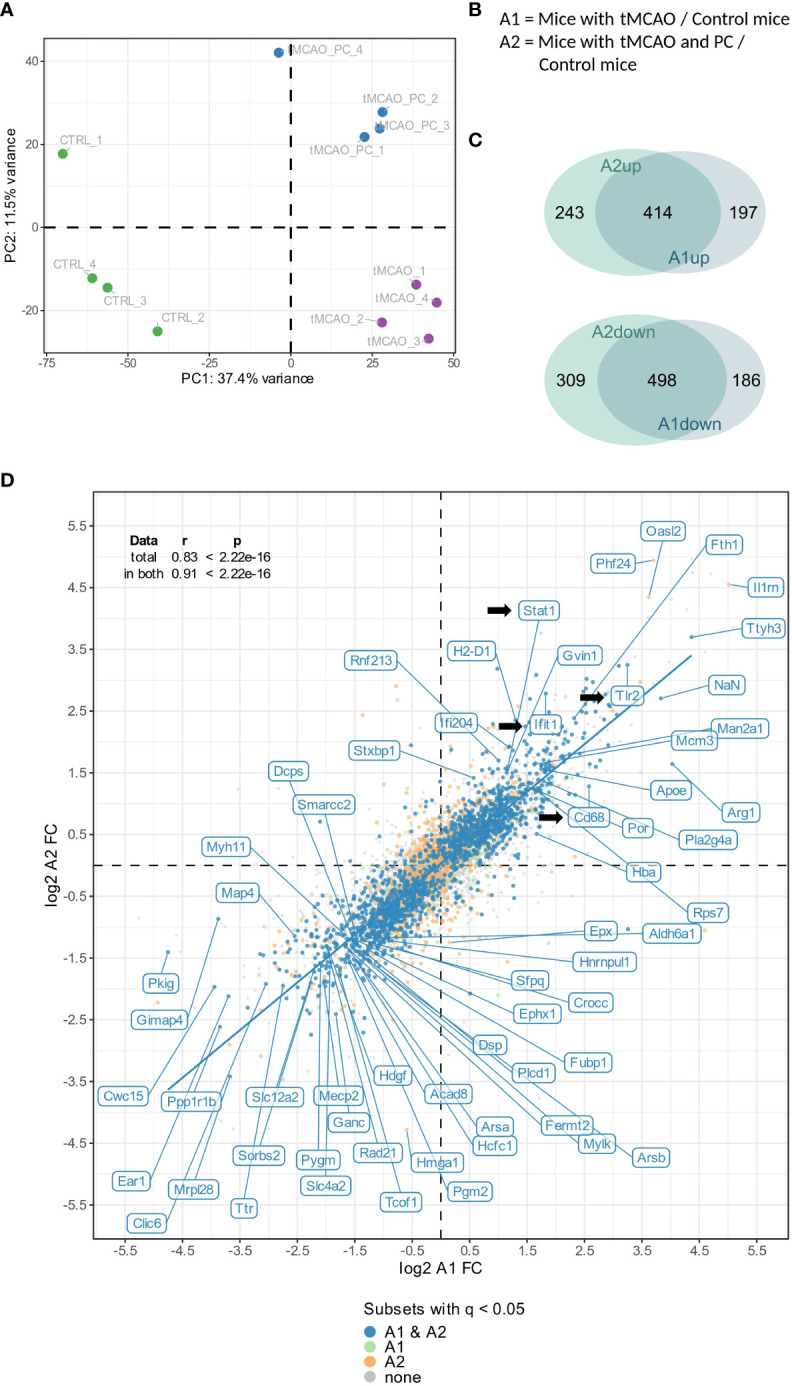
Global microglial proteome changes in response to tMCAO and tMCAO with preconditioning. **(A)** Principal component analysis of the total proteome of non-preconditioned microglia, 2 days after tMCAO (A1) and LPS-preconditioned microglia 2 days after tMCAO (A2) (n=4, each). **(B)** Definition of the relevant comparisons for subsequent differential abundance and enrichment analyses. **(C)** Venn diagrams illustrating the number of proteins being significantly (│log2 FC│≥ 0.58, meaning 50% change and q-value ≤ 0.05) either up- (A1up, A2up, upper panel) or downregulated (A1down, A2down, lower panel), either in both datasets (overlapping area) or uniquely in one of both datasets. **(D)** Correlation plot of the log2 FCs of the A1 and A2 comparison. In the upper left corner, the correlation statistics for total as well as significant proteomes are depicted. (As indicated, in both A1 and A2 significantly changed proteins are labelled in blue, in A1 significantly changed proteins are labelled in light green, in A2 significantly changed proteins are labelled in orange and non-significant proteins are colored in gray. DEPs are labelled with their respective name, if for one of both comparisons │log2 FC│> 3.5 and the q-value < 0.05, or if │log2 FC│> 1.2 and q-value < 10-9; Log2FC, log2 fold change; In addition, Cd68, Ifit1 and Tlr2 are labelled.).

To categorize global proteomic changes in microglia induced by focal brain injury and inflammatory preconditioning, a principal component analysis was performed on the total proteomes of individual microglial cells isolated from three experimental cohorts (naïve microglia, microglia from brains after tMCAO, microglia from brains after tMCAO with previously induced LPS preconditioning). A clear separation and clustering by tMCAO alone, as well as by preconditioning and tMCAO, was found (**Figure 3A**). Furthermore, all tMCAO samples were separated along the first principal component (explaining ~ 37.4% of the total variance) from untreated non-tMCAO samples (CTRL), indicating that tMCAO in general induces similar characteristic changes in microglia, independent of preconditioning. Nevertheless, 11.5% of sample variation is explained by the second principal component, wherein a clear separation is visible between preconditioned tMCAO (tMCAO_PC) and non-preconditioned tMCAO microglia (tMCAO); suggesting specific additional changes within microglial proteome induced by preconditioning. As such, we performed a differential abundance analysis to identify the proteomic changes commonly induced by tMCAO and by tMCAO with preconditioning (tMCAO versus CTRL and tMCAO_PC versus CTRL, abbreviated with A1 and A2, respectively, **Figure 3B**). We also performed comparative analyses of up- or downregulated proteins in A1 and A2 to specifically identify the differentially regulated proteins induced by preconditioning. While the majority of up- or downregulated proteins were shared (414 upregulated, 498 downregulated) between the A1- and A2- comparisons, a substantial amount were significantly altered when affected mice received LPS preconditioning before tMCAO (243 upregulated, 309 downregulated, **Figure 3C**). This is also reflected in the correlation plot (**Figure 3D**; **Supplementary Figures 2A, B**), where a strong correlation between changes in A1 and A2 was identified both for the whole proteome (Spearman’s ρ = 0.83) and for proteins identified as significantly changed in both comparisons (q-value ≤ 0.05, Spearman’s ρ = 0.91). Therefore, the generalized proteomic response of non-preconditioned and preconditioned microglia to tMCAO is generally similar (**Figure 3D**). Among the significantly up- and downregulated proteins are several candidate proteins associated with inflammation, as well as restriction of inflammation. Notably, TLR2, a critical receptor for the microglial response to ischemia (62), was among the top 10 upregulated proteins in comparisons A1 and A2 (**Figure 3D**; **Supplementary Figures 2A, B**). Several other interferon related proteins (e.g., STAT1, Ifit1) appeared to be upregulated, particularly in preconditioned microglia (**Figure 3D**; **Supplementary Figures 2A, B**). Moreover, the lysosomal protein CD68 (63) was more upregulated after preconditioning (log2 fold change (Log2FC) A2 = 2.57 vs. Log2FC A1 = 1.27). Amongst the most downregulated genes in preconditioned microglia was Mef2c, a factor downregulated in microglia due to the presence of Type I IFN, e.g., during aging (53) (Log2FC A2 = -3.11, **Figure 3D**), but was not identified in non-preconditioned microglia. A large number of proteins related to splicing and histones were also downregulated in both comparisons (**Figure 3D**; **Supplementary Figures 2A, B**).

### KEGG pathway and gene ontology enrichment analysis revealed changes in immune response, metabolism, debris clearance and transcriptional/translational processes

The protein lists derived from Venn diagrams were subjected to gene ontology enrichment and KEGG pathway enrichment analysis using DAVID (47), to identify and differentiate specific biological processes that are either generally changed by tMCAO, or only changed by preconditioning. (The complete data set of the KEGG pathway enrichment analysis can be found in **Supplementary Table 2**).

**Figure 4:**
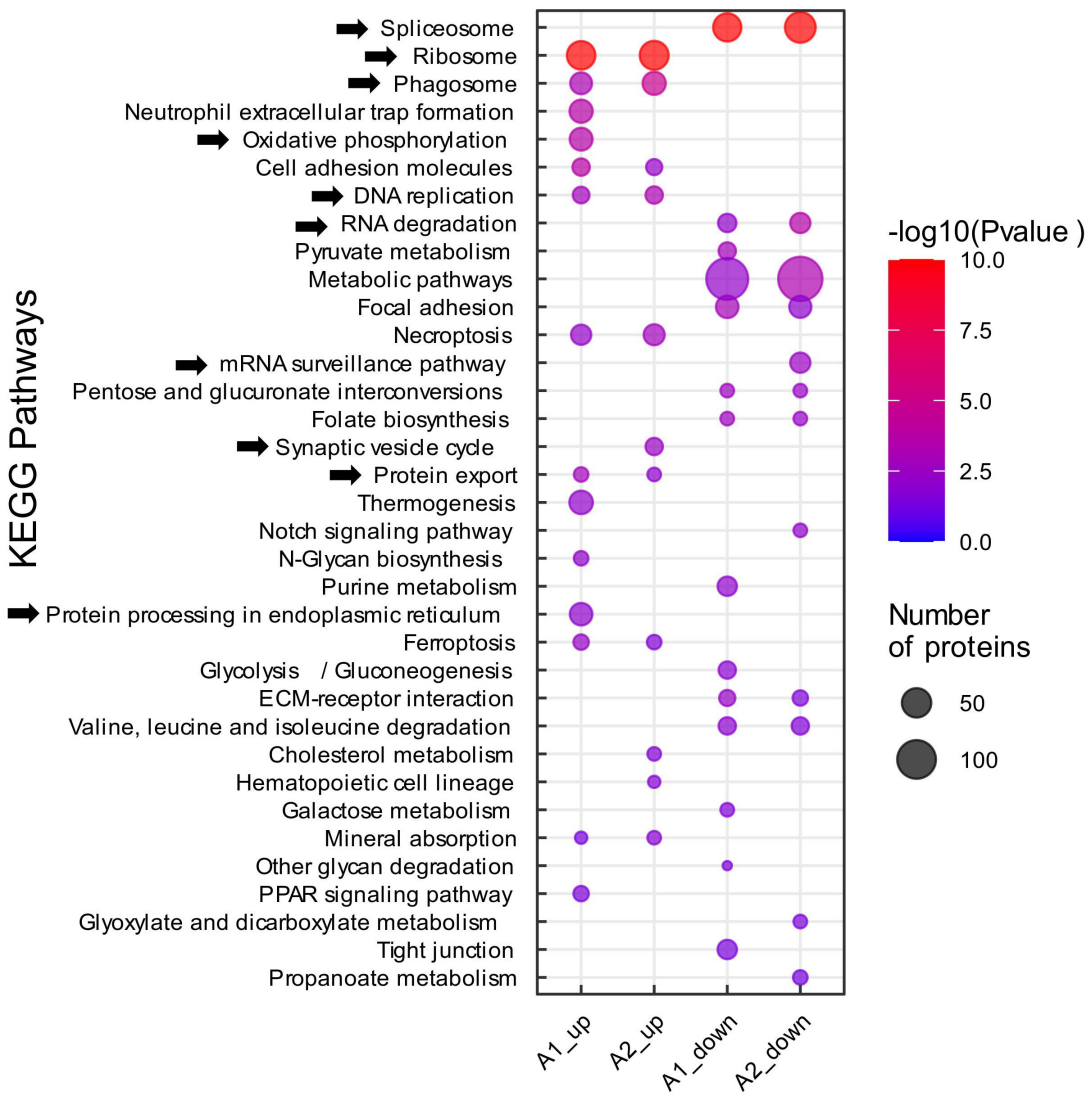
KEGG pathway enrichment analysis. Functional pathway analysis of DEPs with KEGG pathway enrichment analysis. Analyses were performed on proteomic data obtained from non-preconditioned microglia, 2 days after tMCAO (A1) and LPS-preconditioned microglia 2 days after tMCAO (A2). All DEPs (│log2 FC│≥ 0.58 and q-value ≤ 0.05) were submitted to the DAVID platform for KEGG pathway enrichment analysis. The subsequent list was filtered for pathways with a Fisher’s Exact p-value ≤ 0.05, which were considered to be significantly enriched. (As indicated, circle size represents the number of proteins associated with the respective enriched pathway, color scale specifies the level of significance. The experimental groups A1/A2_up/down, denoting the up-and downregulated groups from the single comparisons tMCAO vs. control and tMCAO_PC vs. control, and thereafter the protein lists derived from the Venn diagrams, are depicted on the x-axis).

Amongst the most prominent regulated KEGG pathways were terms related to mRNA processing. Specifically, the “spliceosome” and “RNA degradation” pathways were enriched in the protein lists of downregulated proteins in both non-preconditioned and preconditioned microglia (A1 and A2, respectively) – with more proteins significantly associated with both terms in preconditioned microglia (56 proteins in A2 versus 45 proteins in A1), indicating potentially stronger downregulation (**Figure 4**). The “mRNA surveillance” KEGG pathway was uniquely enriched in the list of downregulated proteins in preconditioned microglia, suggesting a possible alteration of alternative splicing and RNA processing events upon LPS preconditioning. Furthermore, proteins belonging to the ribosomal machinery and DNA replication were upregulated in A1 and A2, alluding to microglial activation associated with cytokine production and secretion after focal ischemia. The KEGG terms “protein export” and “protein processing in endoplasmic reticulum” were also upregulated in both conditions, reinforcing the notion that ribosomal proteins are upregulated, as cytokine production is induced in response to tMCAO and cytokines are often post-translationally modified prior to export and secretion (64) (**Figure 4**). As expected, the KEGG pathway “phagosome” was strongly enriched in the list of upregulated proteins of microglia under both conditions, reflecting the phagocytic activity of microglia after ischemic stroke (65) (**Figure 4**). Slightly more phagosome DEPs were found in preconditioned microglia (26 vs. 22 in non-preconditioned microglia), whilst 9 phagosome DEPs were found to be uniquely upregulated in preconditioned microglia. Of interest, several synaptic proteins were highly upregulated in preconditioned microglia, represented by the KEGG term “synaptic vesicle cycle”, indicating neuron/synapse phagocytosis of activated microglia (**Figure 4**); the Pathview visualization (51) shows an apparent induction of phagocytosis-associated proteins at all levels - uptake, maturation and processing - in both microglia sets, with several proteins either only or more strongly upregulated in preconditioned microglia (**Supplementary Figure 3A**). We also retrieved a list of proteins annotated to be localized to phagocytic vesicles (GO:0045335 “Phagocytic vesicle”) as a proxy for phagosome content. Heatmap visualization and quantification of protein intensities of all mapped phagocytic vesicle proteins, showed that the majority of phagocytic vesicle proteins were upregulated after tMCAO in both microglia sets, with more proteins induced in preconditioned microglia (**Supplementary Figures 3B, C**).

The “oxidative phosphorylation” KEGG pathway was uniquely enriched in non-preconditioned microglia. (**Figure 4**). However, Pathview and heatmap visualizations showed the majority of oxidative phosphorylation associated proteins were also upregulated in preconditioned microglia, at lower levels (**Supplementary Figures 4A, B**); confirmed by quantification of protein intensities of all mapped oxidative phosphorylation proteins (**Supplementary Figure 4C**). Although iNOS was not detected in this proteomics experiment, NADPH oxidase Nox2 – the other major reactive oxygen species (ROS) producing enzyme in microglia – was markedly upregulated in non-preconditioned microglia (Log2FC A1 = 1.76, q-value = 0.0009), whereas it was about 50% less upregulated in preconditioned microglia (Log2FC A2 = 0.85, q-value = 0.0009) (**Supplementary Figure 4D**). Overall, the KEGG pathway enrichment analyses performed indicate biological changes in the microglial response to tMCAO and preconditioning generally similar, but subsequently “fine-tuned”, in terms of greater or lesser induction of key microglial effector functions/phenotypes after tMCAO.

### GO analysis reveals remodeling of microglial inflammatory and functional response to tMCAO with preconditioning

**Figure 5:**
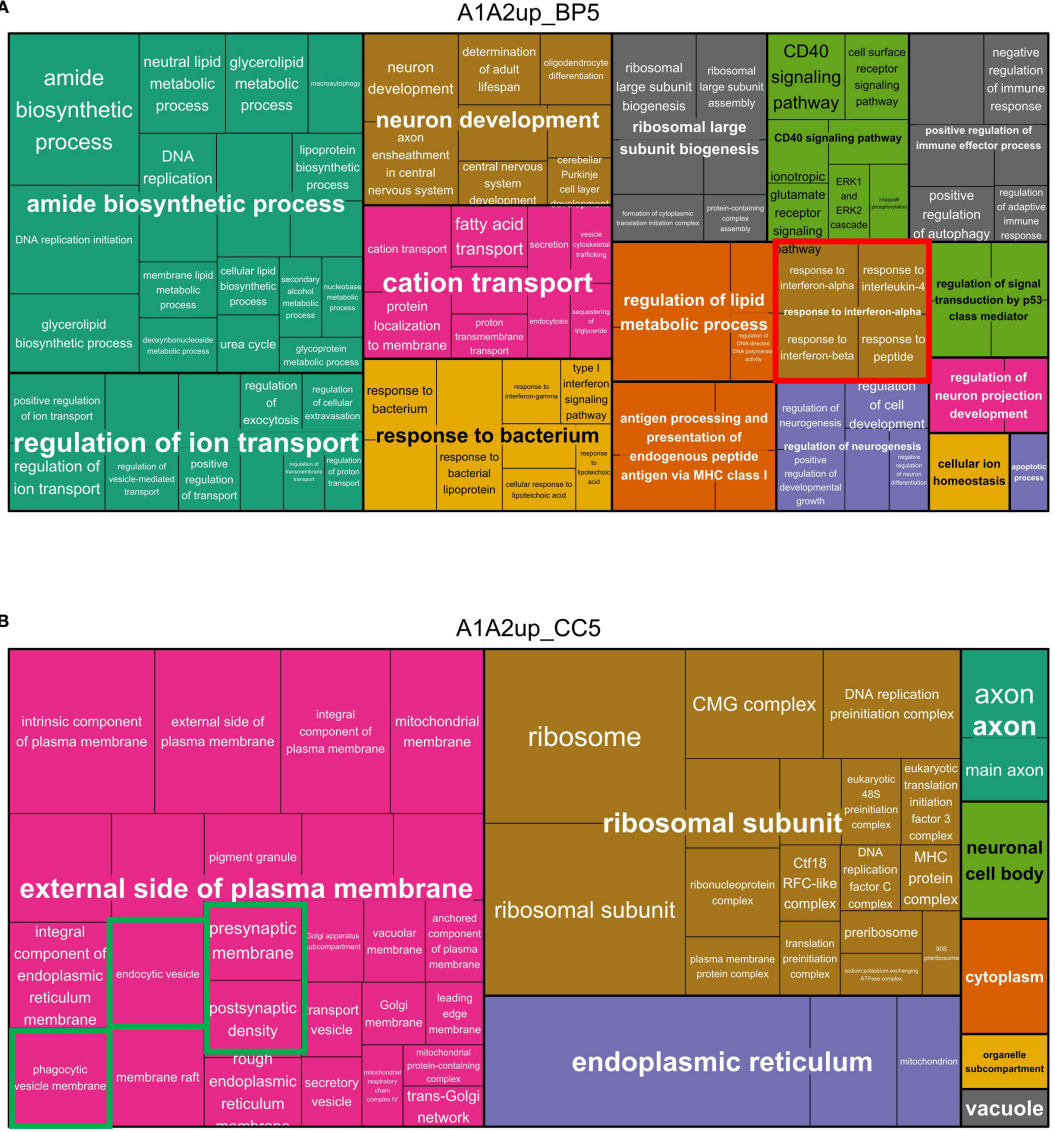
Treemaps of biological process GO-terms enriched in with tMCAO and tMCAO_PC upregulated microglia proteins. **(A)** Level 5 GO-terms of biological processes (BP) enriched in the lists of proteins upregulated in both comparisons (A1&A2) are visualized within a treemap generated with Revigo. Semantically different clusters are coloured differentially. The cluster size is a function of the Fisher’s Exact p-values of the respective enriched GO-clusters and GO-terms: if the p-value is smaller, the cluster size is bigger. All GO-terms were filtered for a cut-off of Fisher’s Exact p-value ≤ 0.1. **(B)** Level 5 GO-terms of cellular components (CC) enriched in the lists of proteins upregulated in both comparisons (A1&A2) are visualized within a treemap generated with Revigo. Semantically similar clusters are coloured differentially. The cluster size is a function of the Fisher’s Exact p-values of the respective enriched GO-clusters and GO-terms: if the p-value is smaller, the cluster size is bigger. All GO-terms were filtered for a cut-off of Fisher’s Exact p-value ≤ 0.1.

While KEGG pathways often contain biologically simplified pathways, GO-terms can provide more detailed biological insights by describing molecular functions, biological processes or cellular components associated with enriched proteins (66). Using the DAVID platform (47), additional enrichment analyses for level 5 biological process and cellular component gene ontology terms were conducted. The enrichment results were subjected to semantic similarity analysis, clustering and visual dimensionality reduction analysis (49). GO enrichment analyses for biological processes revealed two large GO-clusters containing metabolic and RNA/nucleotide and cytoskeleton related changes, primarily found in the lists of downregulated proteins (**Figure 5**), concordant to the KEGG pathway enrichment related changes (**Figure 4**). Concomitantly, cellular component GO-terms, enriched in the list of downregulated proteins, were nearly all related to the spliceosome, chromatin and cytoskeletal components (**Figure 5B**). Several enriched GO-term clusters were detected in different lists of upregulated proteins related to inflammatory activation, vesicle transport pathways such as phagocytosis and endocytosis, and oxidative stress (**Figure 5**). Correspondingly, upregulated proteins mapped or were enriched in cellular component GO-terms related to the respiratory chain and mitochondrial proteins, in addition to vesicles such as the endosome and neuronal and synapse-associated proteins – all indicative of phagocytosis of neuronal debris by microglia (**Supplementary Figure 5**).

Notably, for almost all generally induced GO-term clusters functionally related with tMCAO, more GO-terms were always found to be enriched in the in A2 upregulated protein list, or uniquely present in the A2 upregulated protein list. The opposite can be observed for proteins associated with oxidative stress. These data suggest a fine-tuning of the microglial response by enhancing or attenuating the upregulation of proteins induced by tMCAO in general (A1) (Bar plots of the corresponding clusters, **Figure 4**; **Supplementary Figure 5**).

To investigate the upregulated GO-terms indicating biological process as well as cellular component in more detail, the different semantic similarity clusters were plotted in treemaps. Among the different clusters related to inflammatory activation, a specific cluster of GO-terms enriched in the list of proteins upregulated in A1 and A2 was related to cytokine responses: (i)”response to interferon-alpha”, (ii) “response to interferon-beta”, (iii) “response to type I interferon” and in general (iv)”cellular response to cytokine stimulus”, signifying a potential importance of type I IFN [Interferon-alpha and Interferon-beta (67)] in modifying microglial activation after tMCAO (**Figure 5A**).

Furthermore, cellular component GO-terms enriched in upregulated proteins of non-preconditioned (A1) and preconditioned (A2) microglia were related to mitochondrial proteins, vesicle proteins (especially phagocytic and endocytic vesicle membranes), ribosomal proteins and axonal/synaptic proteins (**Figure 5B**). Plotting the GO-terms enriched uniquely in upregulated proteins of preconditioned microglia, largely resembles the pattern of the enriched GO-terms of both A1 and A2 upregulated proteins (**Supplementary Figures 6**, **7A, B**), supporting the assumption that LPS-induced preconditioning modifies ischemia-dependent proteome response of microglia and related biological effector functions.

### Preconditioning alters I/R-associated microglial inflammatory phenotype via Type I IFN signaling

The conducted KEGG pathway enrichment and gene ontology enrichment analyses indicated substantial inflammatory activation of microglia after tMCAO, irrespective of precedent treatment (**Figure 5**). Considering that microglia can adopt pro- and anti-inflammatory phenotypes after I/R, proteomes form LPS- non-preconditioned and preconditioned microglia were compared with the proteomic core signatures of pro- and anti-inflammatory microglia activation states, as determined previously (52). The detected overlap of proteins revealed a profound induction of a large set of pro- and anti-inflammatory signature proteins significantly changed in tMCAO and tMCAO_PC microglia (**Supplementary Figures 8A, B**). Quantification of the pattern of upregulated DEPs overlapping to either pro- or anti-inflammatory signature proteins, unveiled markedly higher protein intensities of both signatures owing to tMCAO, but increased upregulation after preconditioning (**Supplementary Figures 8A, B**). Specifically, the heatmap of proinflammatory marker proteins shows three separated clusters: (i) a small cluster of proteins downregulated in microglia with tMCAO, (ii) a larger cluster of proteins quite similarly upregulated in tMCAO and tMCAO_PC microglia, (iii) an additional cluster of proteins of markedly stronger upregulation in preconditioned microglia (**Supplementary Figures 8A, B**). Proteins belonging to the latter were Ifih1, Gvin1, Rnf213, Bst2, Ifit1, Parp9, Ifit3, Gbp2, Cmpk2, Isg15, Pyhin1, Marcksl1, Tap2 and Sqstm1.

A heatmap of anti-inflammatory proteins with similar clustering, showed the third cluster of proteins was markedly larger compared to that found in the heatmap of proinflammatory proteins (**Supplementary Figure 8A**). Proteins belonging to that cluster were Pyhin1, Irgm1, Ifit3, Iigp1, Gvin1, Oasl2, Ifit1, Parp9, Ifih1, Gbp2, Cmpk2, Isg15, Parp14, Dhx58, Trex1, Stx18 and Sqstm1. These proteins belong to clusters of proteins more strongly induced in preconditioned microglia and are either part of the cellular type I IFN response or modulate type I IFN signaling. Hence, data support the assumption that inflammatory activation and induction of microglial pro- and anti-inflammatory signature proteins are likely driven and characterized by type I IFN induction, which appears to be enhanced in LPS-preconditioned microglia.

**Figure 6:**
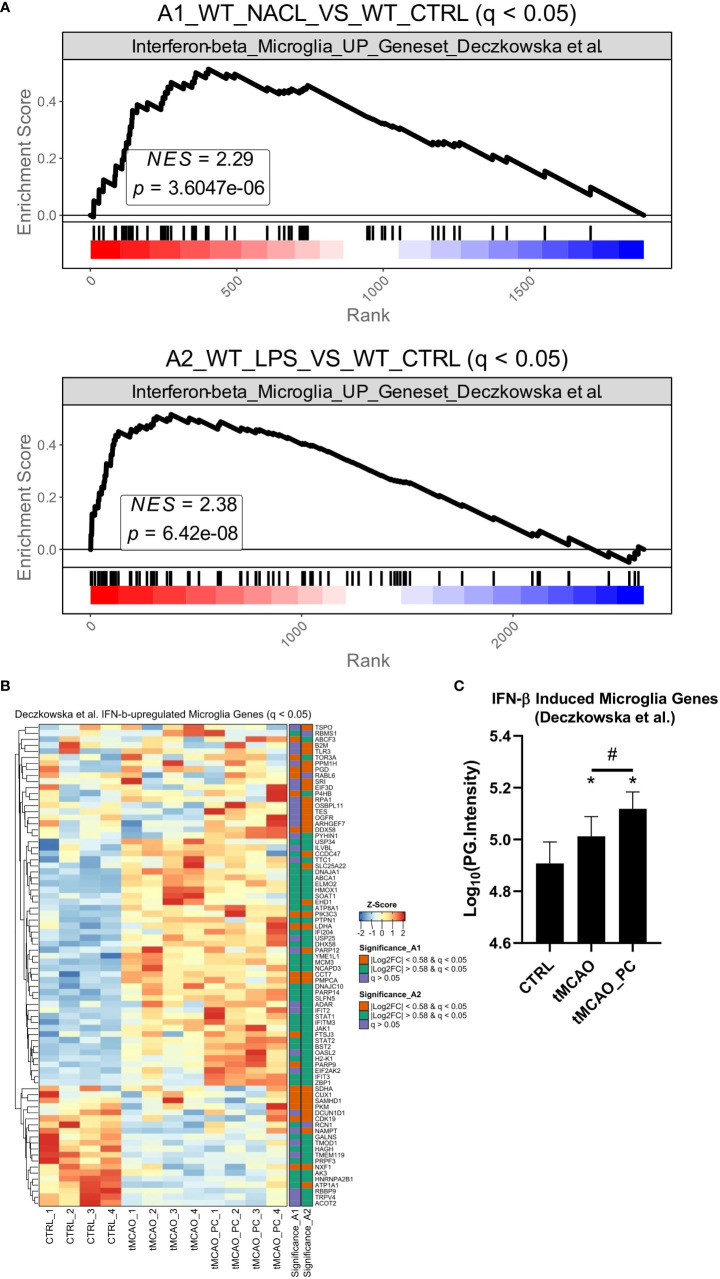
tMCAO-related microglial inflammatory activation resembles a type I IFN-signature. **(A)** Gene set enrichment analyses for all by IFN-b in microglia induced genes [Geneset from (53)] Upper plot: Visualization and statistics of the enrichment of microglia interferon signature in the A1 comparison. Lower plot: Visualization and statistics of the enrichment of microglia interferon signature in the A2 comparison. **(B)** Heatmap visualization of the z-score normalized protein intensities of all samples Only proteins are shown that were significantly changed in A1 or A2 (q ≤ 0.05) and belong to the IFN-β induced gene signature (53) (n=4, each). On the right side of the heatmap an annotation is shown as to whether a protein was significantly changed in A1 or A2 and if yes, above or below the fold change cut-off of │log2 FC│≥ 0.58. **(C)** Bar charts of Log10(PG.Intensities) of all in A1 or A2 significantly changed proteins belonging to the IFN-β induced gene signature (n=80 proteins, a Friedman test was conducted, with a p-value = 0.0001 with *Post-hoc* Wilcoxon signed-rank tests with * = p < 0.05 for comparisons to the control group, specifically p = 0.0022 for CTRL vs. tMCAO, p = 0.0003 for CTRL vs. tMCAO_PC and # = p < 0.05 for the comparison tMCAO to tMCAO_PC, specifically p = 0.0026). All p-values were adjusted for multiple testing with Bonferroni-Holm correction.

A gene set enrichment analysis (GSEA) was performed on both proteomic datasets to provide further evidence, using a RNAseq analysis dataset obtained from adult microglia after *in vivo* IFN-β treatment (53). Those genes upregulated by IFN-β were used to perform a GSEA with our two datasets. Significant enrichment of the type I IFN microglia signature was observed in tMCAO-microglia proteomes and a slightly stronger enrichment in the preconditioned microglia. Heatmap visualization and quantification of the protein intensities of IFN-β dependent proteins showed that the majority is more strongly upregulated in the preconditioned microglia (**Figures 6A–C**) –data that substantiate a significant impact of type I IFN on tMCAO induced and by LPS preconditioning modified microglial proteome patterns.

**Figure 7:**
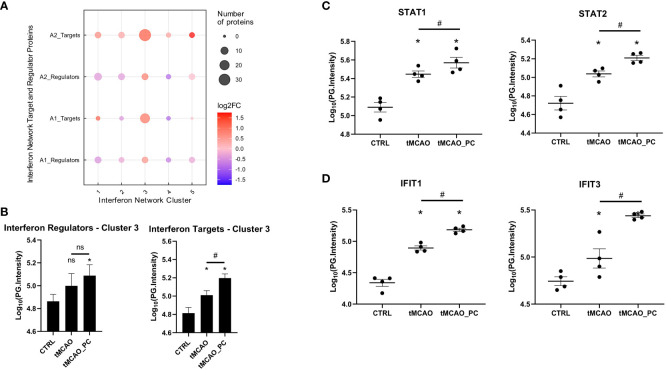
The type I IFN microglial signature after tMCAO is likely driven by STAT1/2. **(A)** Mapping of significantly changed proteins to the IFN-network of regulators and interferon target genes as determined previously by (56). **(B)** Bar charts of Log10(PG.Intensities) of all in A1 or A2 upregulated significantly changed proteins belonging to cluster 3 IFN-regulators (left plot), or cluster 3 IFN-targets (right plot) (n=7 proteins for IFN-regulators, n=32 proteins for IFN-targets. A Repeated measures ANOVA was conducted, with a p-value = 0.0401 for regulator and p < 0.0001 for target proteins. *Post-hoc* Holm-Sidak tests were conducted with * p < 0.05 for comparisons to the control group and # p < 0.05 for the comparison tMCAO to tMCAO_PC, as indicated in the respective figure. Specifically, p = 0.0397 for CTRL vs. tMCAO_PC for regulator proteins and for target proteins p < 0.0001 for all 3 comparisons). **(C, D)** Bar chart scatter plots of Log10(PG.Intensities) of STAT1 and STAT2, belonging to cluster 3 IFN-regulators **(C)** and Ifit1, Ifit3, representing cluster 3 IFN-targets **(D)**, as stated previously (56) (n=4, each). Shown are the FDR-adjusted p-values (=q-values) as reported by Spectronaut software in the differential abundance analysis; *q ≤ 0.05 for comparisons to the control group and # q ≤ 0.05 for the comparison tMCAO to tMCAO_PC. Specifically, q-values for STAT1 were q = 0.0473 for tMCAO vs. CTRL, q = 8.011E-14 for tMCAO_PC vs. CTRL and q = 0.00016 for tMCAO_PC vs. tMCAO. q-values for STAT2 were q = 0.0289 for tMCAO vs. CTRL, q = 0.0084 for tMCAO_PC vs. CTRL and q = 0.0156 for tMCAO_PC vs. tMCAO. q-values for Ifit1 were q = 0.00056 for tMCAO vs. CTRL, q = 3.7663E-05 for tMCAO_PC vs. CTRL and q = 0.00059 for tMCAO_PC vs. tMCAO. q-values for Ifit3 were q = 0.001 for tMCAO vs. CTRL, q = 5.4312E-05 for tMCAO_PC vs. CTRL and q = 5.3175E-07 for tMCAO_PC vs. tMCAO.

Type I IFN signaling is highly diverse, involving multiple pathways and co-regulated protein networks designed to provide robust host defense without inducing autoimmunity (67). Meanwhile, a detailed description of different evolutionarily conserved interferon network clusters has been published (56). These contain both regulator proteins and target proteins [i.e., interferon signature genes (ISG)] and were obtained and mapped to the tMCAO-associated microglia proteome data. Visualization of the mapped network clusters shows a distinct enrichment of cluster 3 (C3) (**Figure 7A**). Both regulator and target proteins of cluster 3 are upregulated in tMCAO-associated microglia. In preconditioned microglia, considerably more C3 target proteins were upregulated (13 proteins in A1; 24 proteins in A2) compared to non-preconditioned microglia (**Figure 7A**). Quantification of all C3 regulator and target protein intensities showed significant upregulation of C3 regulator proteins in preconditioned microglia (**Figure 7B**). Furthermore, C3 target proteins increased their protein intensities in non-preconditioned microglia subjected to tMCAO, alongside a markedly increased upregulation in preconditioned microglia (**Figure 7B**).

The major C3 regulators were studied to more specifically verify effects of tMCAO and microglial preconditioning on C3 interferon network activit. While Irf9 was not identified, Irf7 was unaltered. In contrast, STAT1 and STAT2, the main constituents of the interferon stimulated gene factor 3 (ISGF3) transcription factor complex (56), were markedly induced in tMCAO-associated microglia. Intriguingly, a stronger induction in preconditioned microglia was verified (**Figure 7C**), while two representative members of Interferon-induced protein with tetratricopeptide repeats (IFIT) protein family – Ifit1 and Ifit3 – and thus prominent C3 target proteins, showed increased upregulation in LPS-preconditioned microglia (**Figure 7D**). Thus, the significant effect of type I IFN on I/R-induced and LPS-preconditioned microglial proteome patterns is likely driven by the cluster 3 regulators STAT1/2.

In order to substantiate proteomic findings on the impact of preconditioning on alteration of I/R-associated microglial inflammatory phenotype via Type I IFN signaling, targeted *in vitro* studies on microglial BV2 cells and immunohistochemical measurements of microglial STAT1 expression in brain sections were performed. As shown in **Table 2**, priming of microglial BV2 cells with LPS as well as their stimulation with OGD induced a quite similarly increased gene expression of IFN-α (p < 0.05), whereas IFN-β expression was merely increased by ODG stimulation in LPS-primed cells (p < 0.05). Accordingly, the gene expression of STAT1 and STAT2 were similarly increased after LPS priming and OGD stimulation, respectively (p < 0.05). Remarkably, OGD stimulation of LPS-primed BV2 cells elicited significantly greater expression of STAT1 and STAT2 (p < 0.05). Regarding gene expressions of pro- and anti-inflammatory cytokines, it was found that, as expected, LPS priming led to a significant increase in the expression of the pro-inflammatory IL-1β (p < 0.05). In contrast, IL-10 was significantly more expressed after OGD stimulation and combined LPS/OGD treatment (p < 0.05). Microglial STAT1 expression obtained from Iba1+ cells showed that tMCAO resulted in a significant increase in STAT1 immunoreactivity, whereas LPS-induced preconditioning markedly enhanced it (**Supplementary Figure 9**).

**Table 2:** Gene expression of representative/different INF network members in microglial BV2 cells.

Groups	IFN-α	IFN-β	STAT1	STAT2	IL-1β	IL-10
Control	1.00 (1.00, 1.00)	1.00 (1.00, 1.00)	1.00 (1.00, 1.00)	1.00 (1.00, 1.00)	1.00 (1.00, 1.00)	1.00 (1.00, 1.00)
LPS	2.39 (1.86, 2.63) *	2.03 (1.53, 2.35)	1.96 (1.85, 2.32) *	1.70 (1.52, 1.93) *	1.98 (1.88, 2.20) *	1.40 (1.23, 1.49)
OGD	1.99 (1.44, 2.05) *	1.88 (0.98, 2.20)	1.71 (1.58, 1.98) *	1.37 (1.09, 1.58) *^§^	1.07 (0.96, 1.60)	0.76 (0.34, 1.11) ^§^
LPS + OGD	3.56 (3.19, 4.29) *^#§^	2.60 (2.25, 3.99) *^#^	2.75 (2.49, 3.08) *^#§^	2.13 (2.00, 2.35 *^#§^	1.06 (1.00, 1.41)	1.35 (1.24, 1.74) *^#^

Microglial BV2 cells were cultured for # days/after becoming adherent and then either harvested (Control), primed for # hours by LPS (100 ng/mL) (LPS), stimulated.

by Oxygen-glucose deprivation (OGD) for 3 hours or primed by LPS and subsequently ODG-stimulated (LPS+OGD).

Values are given as medians as well as the first quartile and third quartile in parenthesis; n = 5 per group and treatment, p < 0.05, *significant differences vs. Control,

^§^significant differences vs. LPS, ^#^significant differences vs. OGD within each group.

## Discussion

Microglia are likely the first responders to ischemia, given their ceaseless surveillance of the microenvironment for alterations (68, 69), and undergo dose-dependent reprogramming and metabolic shift as a consequence of TLR4 priming (27, 28, 70). Hence, microglia are viewed as key target cell type for improved insights into the complex molecular reprogramming due to brain I/R and endogenous neuroprotection by PC (71). Brain I/R and PC-related changes in microglial gene expression signatures have been widely described (28, 72–74), but less is known about the extent and functional impact of proteomic alterations in microglia. For the first time, we comprehensively report the effects of LPS-induced PC on microglial proteomic changes induced by transient MCAO in adult mice.

Our data confirm previous cell-specific and genomic reports that preconditioning, which reduces the deleterious consequences of focal brain I/R, is apparently driven by biological processes associated with modified microglial activation and upregulation of specific inflammatory responses that modulate microglial pro-inflammatory activity (74–76). Specifically, an enhanced enrichment of type I IFN in signature was observed in microglial proteomes after preconditioning and subsequent focal brain I/R (**Figure 6**). Parsing the evolutionarily conserved interferon network clusters involved (56), revealed an enhanced upregulation of cluster 3 regulator proteins in preconditioned microglia (**Figure 7**). This interpretation of the proteomic data is supported by the targeted analyzes of the expression data of the cell culture analyzes and brain slice IHC quantifications. The gene expression data showed that LPS preconditioning led to a significant increase in the expression of IFN-α, STAT1 and STAT2, comparable with previous data (24). The consecutive stimulation of LPS-preconditioned microglial BV2 cells displayed an additional increased gene expression. The IHC determined enhanced microglial STAT1 expression in the infarct core of LPS-preconditioned mice supports the proteome findings as well. Therefore, these findings prove on the translational level that the previously suggested type I IFN signaling in microglia does play a critical role in multiple forms of preconditioning-mediated protection (74). The advantage of proteomics over other high throughput omics techniques is its assessment of the end product arising from a combination of adaptations on every level. This is particularly relevant in stroke, after which translation is stalled and altered mRNA levels, detected by transcriptomics, may not correlate with similar changes in protein levels (22, 77). Yet, it must be considered that the effects of type I IFNs may be time-dependent: administration of IFNβ shortly before or after tMCAO exerts a protective effect against ischemic stroke, with a significant reduction in infarct volume through its anti-inflammatory properties targeting reperfusion injury, which is observed after a few days and one or three weeks (78–81). Repeated systemic application of IFNβ for three or seven days failed to protect against experimental ischemic brain injury, determined one week after tMCAO in rats. This was associated with significant weight loss and alterations in hematology and chemistry profiles (82). Therefore, early temporal activation of type I IFN signaling system yields resistance to subsequent prolonged ischemic exposure. This temporal pattern sits well with the concept of microglial priming (26, 83, 84) and allows the possibility that downstream microglial pathways, including those involving ISG products, are critical effectors in mediating protection and/or enhanced recovery (74).

To determine the PC-induced modulation of effector mechanisms, or cellular functions, known to be involved in key microglial response patterns to brain I/R, KEGG pathway analyses were performed to examine microglial ROS production and phagocytic activity. Considering that ROS production in microglia underlies three main sources (i) the mitochondrial respiratory chain where ROS accumulate physiologically, (ii) inducible nitric oxide synthase (iNOS) and (iii) cellular NADPH oxidases (NOX), in microglia mainly Nox2 (85, 86), our data showed a markedly reduced upregulation of microglial Nox2 due to PC, which may be associated with the reduction in infarct size by LPS-PC. These results confirm a previous report which showed that LPS-induced PC is accompanied by a reduced infarct size and preserved neurovascular function (87). Phagocytosis is generally considered a beneficial process that leads to clearance of potentially harmful cellular components and may also contribute to the resolution of neuroinflammation after ischemic stroke (36, 88). It has been shown that upregulation of microglial phagocytic activity occurs rapidly after brain ischemia (89) and represents the predominant phagocytic activity (65) for removal of released danger signals resulting from dying cells and debris after necrotic cell death. Type I IFNs also act directly on microglia to modulate phagocytosis, including the clearance of degenerating axons, degraded myelin and apoptotic cells (90, 91), suggesting intimate interplay between LPS- PC-induced modulation of type I IFN signaling and enhanced microglial phagocyte activity.

Still, some limitations must be considered – as we used the entire affected hemisphere for microglial isolation, no differential proteome analysis could be performed for infarct core, penumbra and surrounding brain tissue. Furthermore, we used CD11b for cell-sorting of microglial cells from brain tissue. Even when brains have been cleared of intravasal blood, invading monocytes/macrophages as well as subsets of polymorphonuclear neutrophil granulocytes (PMNs) also express the surface integrin CD11b (92–96). However, up to three days after ischemic stroke, invading monocytes/macrophages represent a minority of all CD11b+ cells (65, 97, 98). Schilling et al. showed that there were no infiltrating macrophages on the first day and that the number of infiltrating macrophages remained very low on day 2 after 30 min MCAO (65). Werner et al. showed by lineage tracing of blood-born macrophages that less than 20% of all Iba1-positive cells represent infiltrating macrophages at day 3 after IS (98). In addition, a recent study revealed that CD11b+ PMNs represent merely about 30% of CD11b+ leukocytes (95). It can therefore be assumed that the majority of observed proteomic changes documented here can be assigned to microglia. As we only examined the effects of LPS-PC at a time point (48h post-tMCAO) at which is known from previous studies that microglia are the most active immunocompetent cells in ischemic brain tissue, follow-up studies characterizing longer-term effects are required.

In summary, we have provided translational evidence that distinct microglial reprogramming, inducing modified proteomic patterns, underlies endogenous tolerance to transient brain ischemia. Multifunctional adaptations such as inflammatory activation, phagocytosis and ROS production occurred in microglial cells in response to I/R, all of which were differentially modulated by LPS-PC. In particular, LPS-PC was characterized by the induction of an evolutionarily conserved type I interferon response, which may be driven mainly by STAT1/2 activation. Our results offer insights into the regulation of the innate immune response to the ischemic/reperfused brain and immunological preconditioning, which can be starting points for further studies aimed at modulating neuroinflammation to induce neuroprotection due to brain ischemia and reperfusion.

## Data availability statement

The datasets presented in this study can be found in online repositories. The names of the repository/repositories and accession number(s) can be found below: http://www.proteomexchange.org/, PXD031973 http://www.proteomexchange.org/, PXD031930.

## Ethics statement

The animal study was approved by Thuringian State Office for Food Safety and Consumer Protection. The study was conducted in accordance with the local legislation and institutional requirements.

## Author contributions

DLH: Conceptualization, Data curation, Formal analysis, Methodology, Visualization, Writing—original draft, Writing—review & editing. FH: Data curation, Formal analysis, Writing—original draft, Writing—review & editing. EC: Data curation, Formal analysis, Writing—original draft, Writing—review & editing. NR: Formal analysis, Writing—review & editing. TTDD: Formal analysis, Writing—review & editing. EKS: Formal analysis, Writing—review & editing. NO: Formal analysis, Writing—original draft, Writing—review & editing. LB: Formal analysis, Writing—review & editing. TL: Methodology, Formal analysis, Writing—original draft, Writing—review & editing. PF: Methodology, Formal analysis, Writing—review & editing. HM: Resources, Writing—original draft, Writing—review & editing. RB: Conceptualization, Data curation, Formal analysis, Methodology, Project administration, Resources, Supervision, Writing—original draft, Writing—review & editing.

## Supplementary material

Supplementary Figure 1 Validation of microglial cell enrichment via MACS. **(A–C)** FACS analysis of MACS-isolated CD11b+ microglia. Different cell suspensions before and after MACS-based separation were stained with an APC-coupled CD11b antibody, followed by FACS analysis. **(A)** Representative FACS profile of the original fraction that still contains all brain cells before MACS separation. **(B)** Representative FACS profile of the non-target cell fraction (NTCF) containing all CD11b- cells after MACS separation. **(C)** Representative FACS profile of the CD11b positive cell fraction (CD11b+) containing all CD11b+ cells, mainly microglia. **(D)** Principal component analysis of proteomes determined for CD11b+ and NTCF fractions showing a clear separation of two clusters. **(E)** Proteins of cells from CD11b+ and NTCF fractions detected and quantified by proteomic analysis, were matched to a brain cell type proteome dataset by (3). Heatmap visualization of all proteins defined to be marker proteins for microglial cells (left plot) and non-microglia cells (right plot). The z-score normalized protein intensities are depicted. A clear pattern of stronger protein intensities of non-microglia proteins within the NTCF samples and microglia proteins within the CD11b+ samples is visible (for both figures n=3 biological replicates sampled from the same mice, one CD11b+/NTCF pair per mouse). **(G)** Quantification of marker proteins for astrocytes, neurons, microglia, and oligodendrocytes found in the microglia-enriched fraction (CD11b+), or the microglia-depleted fraction (NTCF). Microglia marker proteins appear to be strongly enriched within the microglia-enriched fraction, while in the microglia-depleted fraction predominantly astrocyte and oligodendrocyte proteins were detected. **(F)** Dot plot chart of the log10(PG.Intensities) of classical neuronal (Tuj1) and glia marker proteins (Microglia: Iba1/Aif1, Astrocytes: Gfap, Oligodendrocytes: Cnp) in the proteome of CD11b+ and NTCF fractions. Iba1 was detected at high levels in CD11b+ samples only, while for all other marker proteins the opposite applied, i.e. high levels of other brain cell marker proteins were detected only in the NTCF fraction.

Supplementary Figure 2 Correlation plot analysis. Depiction of the Log2 fold changes of the ten most up- and down-regulated proteins in the Depiction of the A1 and A2 comparison.

Supplementary Figure 3 Induction of phagosome proteins and phagocytic vesicle-associated proteins after tMCAO or tMCAO and preconditioning. **(A)** Functional pathway annotation of significantly changed proteins in A1 and A2 to the KEGG pathway: Phagosome with the R package pathview (4). Log2FC of the respective proteins are normalized and shown on a scale between -1 and 1, where positive values indicating significant upregulation are colored red, downregulated proteins are colored blue. On the left part of the respective box with the respective protein name the normalized Log2FC of A1 is displayed; the right half of the respective box with the respective protein name displays the normalized Log2FC of A2. **(B)** Heatmap visualization of the z-score normalized protein intensities of all samples. Only proteins are shown that were significantly changed in A1 or A2 and belong to the GO-term “Phagocytic vesicle” (all samples n=4 biological replicates sampled from different mice). On the right side of the heatmap an annotation is shown as to whether a protein was significantly changed in A1 or A2 and if yes, above or below the fold change cut-off of │log2 FC│≥ 0.58. **(C)** Bar charts of Log10(PG.Intensities) of all in A1 or A2 significantly changed proteins belonging to the GO-term “Phagocytic vesicle” (n=51 proteins, a Repeated measures ANOVA was conducted, with a p-value < 0.0001 with *Post-hoc* Holm-Sidak tests with * = p < 0.05 for comparisons to the control group; specifically p = 0.0005 for CTRL vs. tMCAO, p < 0.0001 for CTRL vs. tMCAO_PC and p = 0.0640 for the comparison tMCAO to tMCAO_PC).

Supplementary Figure 4 Amelioration of ROS-production pathways by preconditioning after tMCAO. **(A)** Functional pathway annotation of significantly changed proteins in A1 and A2 to the KEGG pathway mmu00190: “Oxidative Phosphorylation” with the R package pathview (4). Log2FC of the respective proteins are normalized and shown on a scale between -1 and 1, where positive values indicating significant upregulation are colored red, downregulated proteins are colored blue. On the left half of the respective box with the respective protein name, the normalized Log2FC of A1 is displayed; the right half of the respective box with the respective protein name displays the normalized Log2FC of A2. **(B)** Heatmap visualization of the z-score normalized protein intensities of all samples. Only proteins are shown that were significantly changed in A1 or A2 and belong to the KEGG pathway mmu00190: “Oxidative Phosphorylation” (all samples n=4 biological replicates sampled from different mice). On the right side of the heatmap an annotation is shown as to whether a protein was significantly changed in A1 or A2 and if yes, above or below the fold change cut-off of │log2 FC│≥ 0.58. **(C)** Bar charts of Log10(PG.Intensities) of all significantly changed proteins in A1 or A2 belonging to the KEGG pathway mmu00190: “Oxidative Phosphorylation” (n=69 proteins, a Repeated measures ANOVA was conducted, with a p-value < 00001 with *Post-hoc* Holm-Sidak tests with * = p < 0.05 for comparisons to the control group; specifically p < 0.0001 for both comparisons CTRL vs. tMCAO and CTRL vs. tMCAO_PC and # = p < 0.05 for the comparison tMCAO to tMCAO_PC, specifically p = 0.0424). **(D)** Bar chart of Log10(PG.Intensities) of Nox2 (n=4, each). Shown are the FDR-adjusted p-values (=q-values) as reported by Spectronaut software in the differential abundance analysis; *q ≤ 0.05 for comparisons to the control group and # q ≤ 0.05 for the comparison tMCAO to tMCAO_PC. Specifically, q-values were q = 0.000929 for tMCAO vs. CTRL, q = 0.000903 for tMCAO_PC vs. CTRL and q = 0.0044 for tMCAO_PC vs. tMCAO.

Supplementary Figure 5 Gene ontology enrichment analysis for cellular components. Functional gene ontology enrichment analysis of level 5 GO-terms of cellular components was conducted based on the experimental groups A1/A2_up/down, standing for the up- and downregulated groups from the single comparisons tMCAO vs. CTRL and tMCAO_PC vs. CTRL, and thereafter the protein lists derived from the Venn diagrams. Enriched GO-terms with a Fisher’s Exact p-value ≤ 0.1 were used for semantic similarity clustering – the similarity matrix with specific clusters is depicted in the middle. Each row corresponds to one GO-term and in the matrix each column corresponds to one GO-term. On the left a binary heatmap is displayed showing whether a GO-term was enriched in the protein target list or not, where each row corresponds to one GO-term. On the extreme left, bar plots are shown where cluster-wise the number of GO-terms for each protein list is counted. On the right a word cloud is annotated to the similarity matrix, providing information about the cellular component in keywords, in terms of an overrepresentation of individual words from all GO-terms located in this cluster.

Supplementary Figure 6 Treemaps of biological process GO-terms enriched in with tMCAO and tMCAO_PC upregulated microglia proteins. **(A)** Level 5 GO-terms of biological processes enriched in the lists of proteins upregulated in both comparisons (A1&A2) are visualized within a treemap generated with Revigo. Semantically different clusters are colored differentially. The cluster size is a function of the Fisher’s Exact p-values of the respective enriched GO-clusters and GO-terms: if the p-value is smaller, the cluster size is bigger. All GO-terms were filtered for a cut-off of Fisher’s Exact p-value ≤ 0.1. **(B)** Level 5 GO-terms of cellular components enriched in the lists of proteins upregulated in both comparisons (A1&A2) are visualized within a treemap generated with Revigo. Semantically similar clusters are colored differentially. The cluster size is a function of the Fisher’s Exact p-values of the respective enriched GO-clusters and GO-terms: if the p-value is smaller, the cluster size is bigger. All GO-terms were filtered for a cut-off of Fisher’s Exact p-value ≤ 0.1.

Supplementary Figure 7 Treemaps of biological process GO-terms enriched uniquely in with tMCAO_PC upregulated microglia proteins. **(A)** Level 5 GO-terms of biological processes (BP) enriched in the lists of proteins upregulated uniquely in LPS-primed microglia after tMCAO (A2_up_unique) are visualized within a treemap generated with Revigo. Semantically similar clusters are colored differentially. The cluster size is a function of the Fisher’s Exact p-values of the respective enriched GO-clusters and GO-terms: if the p-value is smaller, the cluster size is bigger. All GO-terms were filtered for a cut-off of Fisher’s Exact p-value ≤ 0.1. **(B)** Level 5 GO-terms of cellular components (CC) enriched in the lists of proteins upregulated uniquely in LPS-primed microglia after tMCAO (A2_up_unique) are visualized within a treemap generated with Revigo. Semantically similar clusters are colored differentially. The cluster size is a function of the Fisher’s Exact p-values of the respective enriched GO-clusters and GO-terms: if the p-value is smaller, the cluster size is bigger. All GO-terms were filtered for a cut-off of Fisher’s Exact p-value ≤ 0.1.

Supplementary Figure 8 Proteomic signature of enhanced inflammatory activation of microglia after tMCAO and preceding LPS-induced inflammatory preconditioning. **(A, B)** Heatmap visualizations of the z-score normalized protein intensities of all samples. Proteins are shown when changed in A1 or A2 (│log2 FC│≥ 0.58 and q-value ≤ 0.05) and belong to the corresponding microglial pro-/anti-inflammatory signature (5) (n=4, each). On the right side of the heatmap an annotation is shown as to whether a protein was significantly changed in A1 or A2 above or below the fold change cut-off of │log2 FC│≥ 0.58. **(C)** Bar charts of Log10 (PG.Intensities, where PG = protein group) of all in A1 or A2 DEPs belonging to either the pro- (upper chart) or anti-inflammatory (lower chart) gene signatures (n=36 proteins for proinflammatory gene signature, n=22 proteins for anti-inflammatory gene signature. A Friedman-test was conducted, with a p-value < 0.05 for both signatures, specifically p < 0.0001 for the proinflammatory signature and p = 0.0002 for the anti-inflammatory signature. *Post-hoc* Wilcoxon signed-rank tests were conducted with *p < 0.05 for comparisons to the control group and #p < 0.05 for the comparison tMCAO to tMCAO_PC. Specifically, p-values for the proinflammatory signature were p = 0.0003 for both CTRL vs. tMCAO and CTRL vs. tMCAO_PC and p = 0.0105 for tMCAO vs. tMCAO_PC. For the anti-inflammatory signature p-values were p = 0.0014 for CTRL vs. tMCAO, p = 0.003 for CTRL vs. tMCAO_PC and p = 0.0462 for tMCAO vs. tMCAO_PC.) All p-values were adjusted for multiple testing with Bonferroni-Holm correction.

Supplementary Figure 9 Effect of temporary focal brain ischemia and 2 days of reperfusion without (tMCAO) or with LPS preconditioning (tMCAO_PC) on microglial STAT1 immunoreactivity. **(A)** STAT1 fluorescence intensity of microglial (Iba1-positive) cells in brains of untreated mice (CTRL), mice 2 days after temporary focal brain ischemia and 2 days of reperfusion without (tMCAO) or with LPS preconditioning (tMCAO_PC). (n=7 for each group, data are given as scatter dot plots, mean ± SEM, *^§^ p < 0.05, * significant differences vs. CTRL, ^§^ significant difference vs. tMCAO. Kruskal-Wallis ANOVA on Ranks, with *Post-hoc* Student-Newman-Keuls test. **(B)** Representative images of microglial cells (left column, green - Iba1 positive), (middle column, brown - STAT1 positive) and overlaid (merge). Note that the fluorescence intensity of Iba1 is markedly increased in animals after tMCAO as a sign of microglial activation and is significantly enhanced in animals after LPS preconditioning and tMCAO. Microglial STAT1 expression is almost absent in control animals, is detectable in animals after tMCAO, and is significantly increased in animals after LPS preconditioning and tMCAO (arrows).
