# Supplementary material for: Impact of inflammatory preconditioning on murine microglial proteome response induced by focal ischemic brain injury
Source: Front Immunol. 2024 Apr 9;15:1227355. doi: 10.3389/fimmu.2024.1227355 (PMC11036884; doi:10.3389/fimmu.2024.1227355)
Supplement: Supplementary Figure 1 — Validation of microglial cell enrichment via MACS. (A–C) FACS analysis of MACS-isolated CD11b+ microglia. Different cell suspensions before and after MACS-based separation were stained with an APC-coupled CD11b antibody, followed by FACS analysis. (A) Representative FACS profile of the original fraction that still contains all brain cells before MACS separation. (B) Representative FACS profile of the non-target cell fraction (NTCF) containing all CD11b- cells after MACS separation. (C) Representative FACS profile of the CD11b positive cell fraction (CD11b+) containing all CD11b+ cells, mainly microglia. (D) Principal component analysis of proteomes determined for CD11b+ and NTCF fractions showing a clear separation of two clusters. (E) Proteins of cells from CD11b+ and NTCF fractions detected and quantified by proteomic analysis, were matched to a brain cell type proteome dataset by (3). Heatmap visualization of all proteins defined to be marker proteins for microglial cells (left plot) and non-microglia cells (right plot). The z-score normalized protein intensities are depicted. A clear pattern of stronger protein intensities of non-microglia proteins within the NTCF samples and microglia proteins within the CD11b+ samples is visible (for both figures n=3 biological replicates sampled from the same mice, one CD11b+/NTCF pair per mouse). (G) Quantification of marker proteins for astrocytes, neurons, microglia, and oligodendrocytes found in the microglia-enriched fraction (CD11b+), or the microglia-depleted fraction (NTCF). Microglia marker proteins appear to be strongly enriched within the microglia-enriched fraction, while in the microglia-depleted fraction predominantly astrocyte and oligodendrocyte proteins were detected. (F) Dot plot chart of the log10(PG.Intensities) of classical neuronal (Tuj1) and glia marker proteins (Microglia: Iba1/Aif1, Astrocytes: Gfap, Oligodendrocytes: Cnp) in the proteome of CD11b+ and NTCF fractions. Iba1 was detected at high levels [file DataSheet_1.pdf]

Figure S1

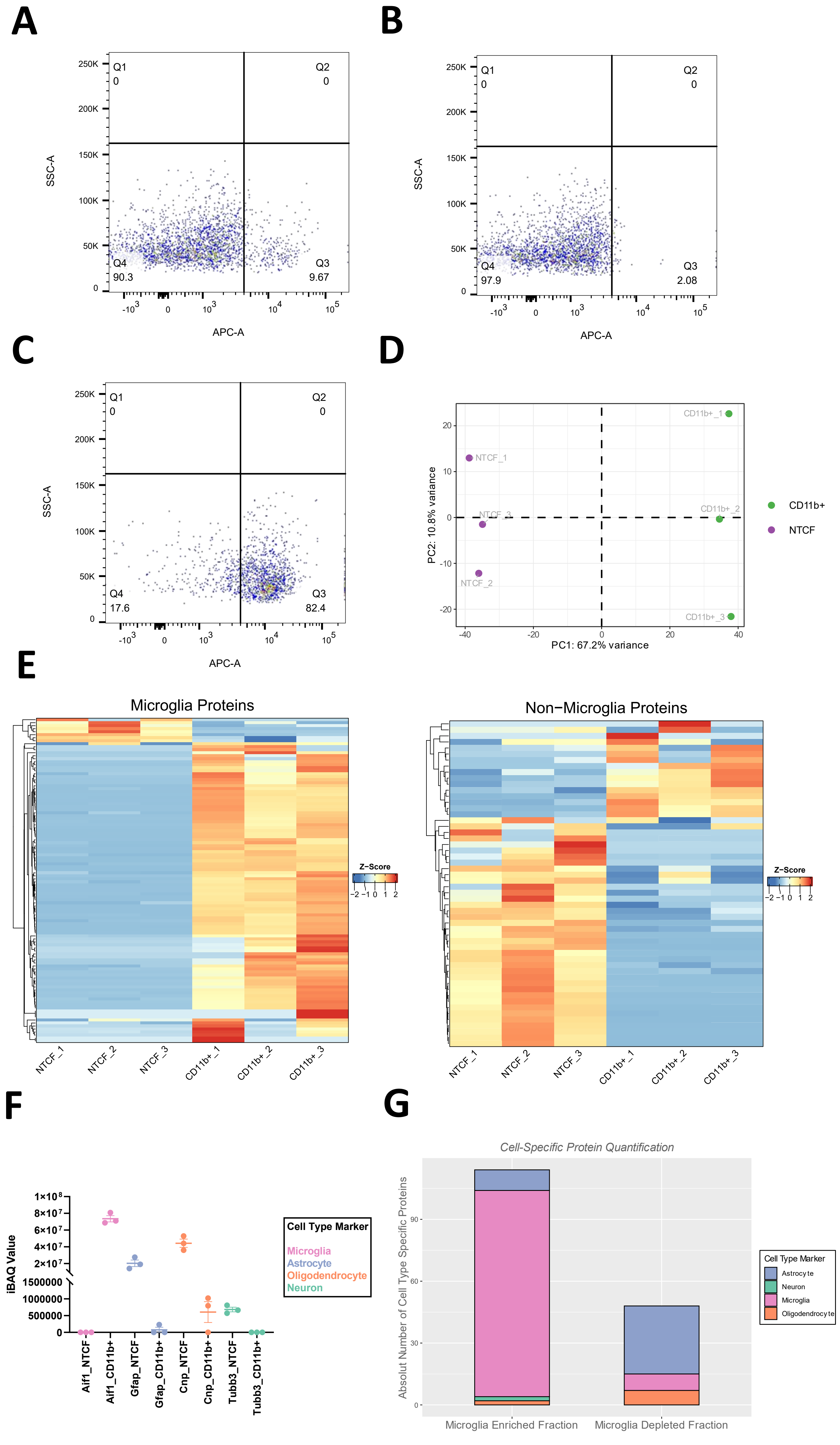
A
B
C
D
E
F
G
Q1
0
Q2
0
Q4
90.3
Q3
9.67
Q4
97.9
Q3
2.08
Q4
17.6
Q3
82.4
SSC-A
APC-A
PC1: 67.2% variance
PC2: 10.8% variance
CD11b+
NTCF
Microglia Proteins
Non−Microglia Proteins
Z-Score
NTCF_1
NTCF_2
NTCF_3
CD11b+_1
CD11b+_2
CD11b+_3
iBAQ Value
Cell Type Marker
Microglia
Astrocyte
Oligodendrocyte
Neuron
Aif1_NTCF
Aif1_CD11b+
Gfap_NTCF
Gfap_CD11b+
Cnp_NTCF
Cnp_CD11b+
Tubb3_NTCF
Tubb3_CD11b+
Cell-Specific Protein Quantification
Absolut Number of Cell Type Specific Proteins
Microglia Enriched Fraction
Microglia Depleted Fraction

Figure S2

A

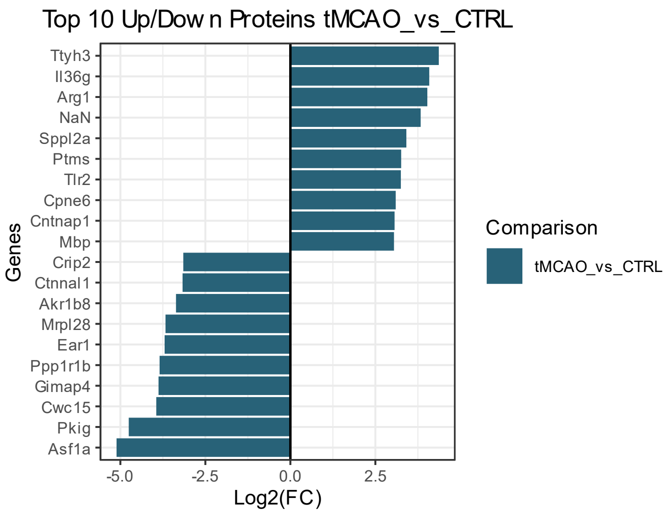

Top 10 Up/Down Proteins tMCAO_vs_CTRL
Ttyh3
Il36g
Arg1
NaN
Sppl2a
Ptms
Tlr2
Cpne6
Cntnap1
Mbp
Crip2
Ctnnal1
Akr1b8
Mrpl28
Ear1
Ppp1r1b
Gimap4
Cwc15
Pkig
Asf1a
Genes
-5.0
-2.5
0.0
2.5
Log2(FC)
Comparison
tMCAO_vs_CTRL

B

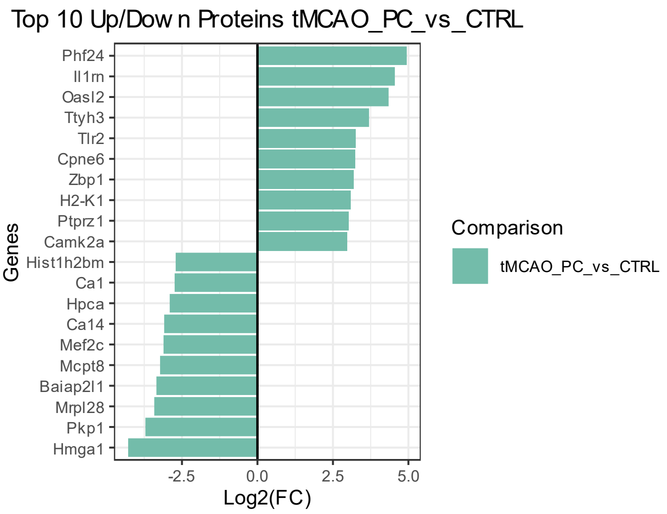

Top 10 Up/Down Proteins tMCAO_PC_vs_CTRL
Phf24
Il1rn
Oasl2
Ttyh3
Tlr2
Cpne6
Zbp1
H2-K1
Ptprz1
Camk2a
Hist1h2bm
Ca1
Hpca
Ca14
Mef2c
Mcpt8
Baiap2l1
Mrpl28
Pkp1
Hmga1
Genes
-2.5
0.0
2.5
5.0
Log2(FC)
Comparison
tMCAO_PC_vs_CTRL

Figure S3

A

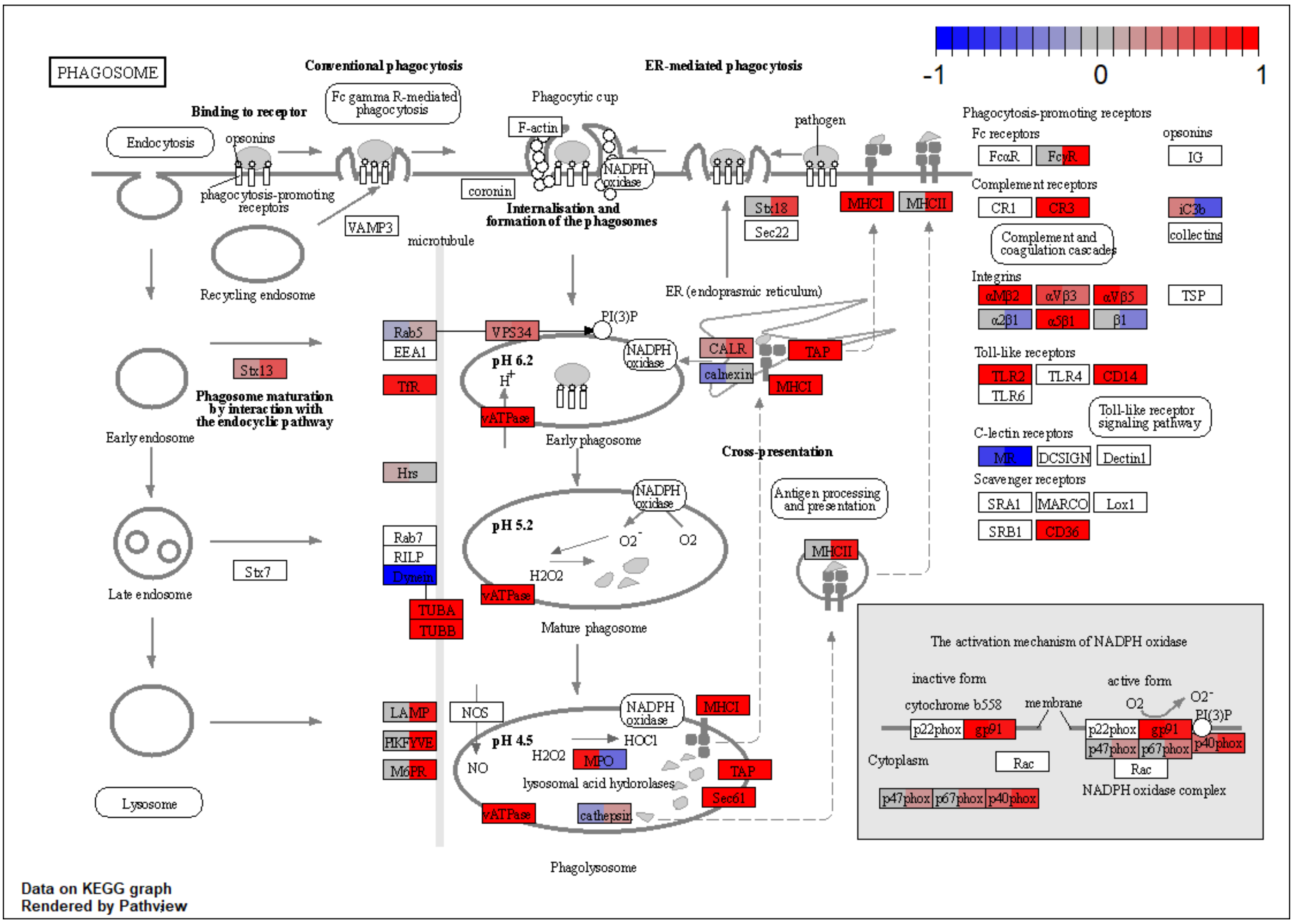

B

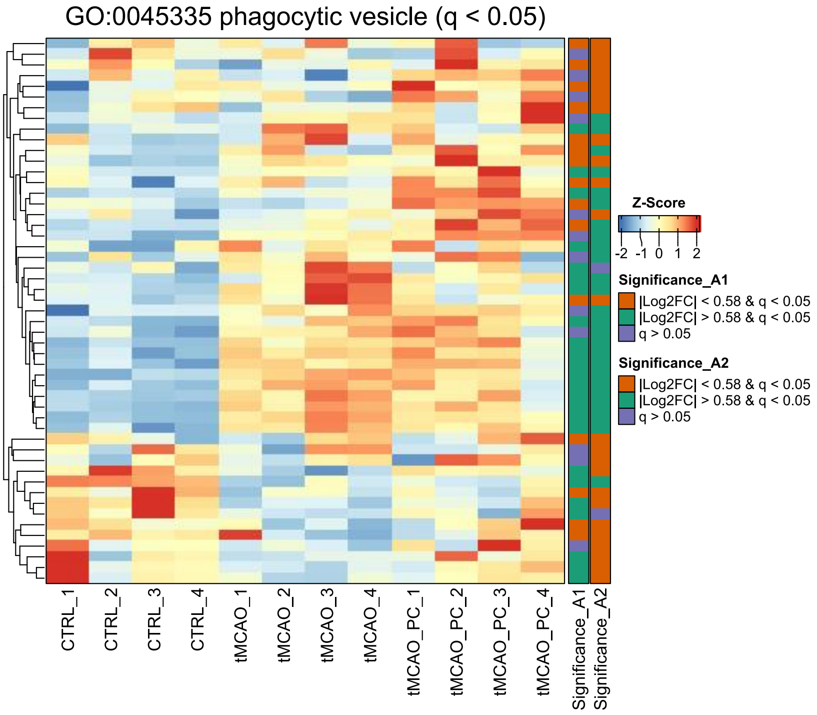

C

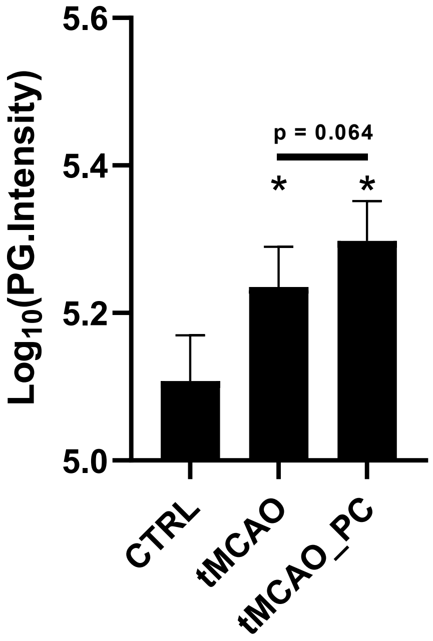

Figure S4

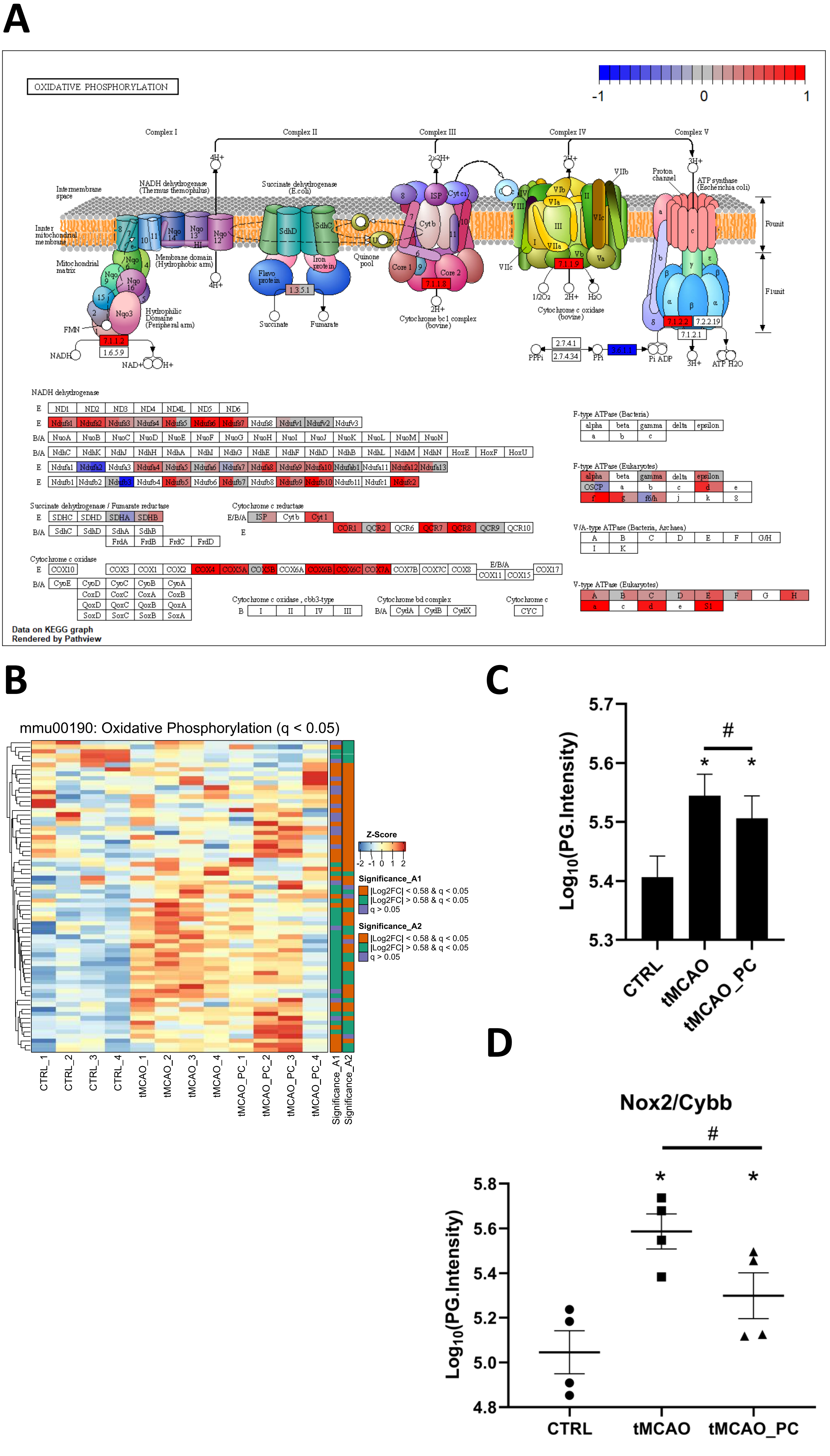

A
OXIDATIVE PHOSPHORYLATION
Complex I
Complex II
Complex III
Complex IV
Complex V
NADH dehydrogenase
Succinate dehydrogenase / Fumarate reductase
Cytochrome c reductase
Cytochrome c oxidase
F-type ATPase (Bacteria)
F-type ATPase (Eukaryotes)
V/A-type ATPase (Bacteria, Archaea)
V-type ATPase (Eukaryotes)
Data on KEGG graph
Rendered by Pathview
B
mmu00190: Oxidative Phosphorylation (q < 0.05)
Z-Score
Significance_A1
|Log2FC| < 0.58 & q < 0.05
|Log2FC| > 0.58 & q < 0.05
q > 0.05
Significance_A2
|Log2FC| < 0.58 & q < 0.05
|Log2FC| > 0.58 & q < 0.05
q > 0.05
CTRL_1
CTRL_2
CTRL_3
CTRL_4
tMCAO_1
tMCAO_2
tMCAO_3
tMCAO_4
tMCAO_PC_1
tMCAO_PC_2
tMCAO_PC_3
tMCAO_PC_4
C
Log10(PG.Intensity)
CTRL
tMCAO
tMCAO_PC
D
Nox2/Cybb
Log10(PG.Intensity)
CTRL
tMCAO
tMCAO_PC

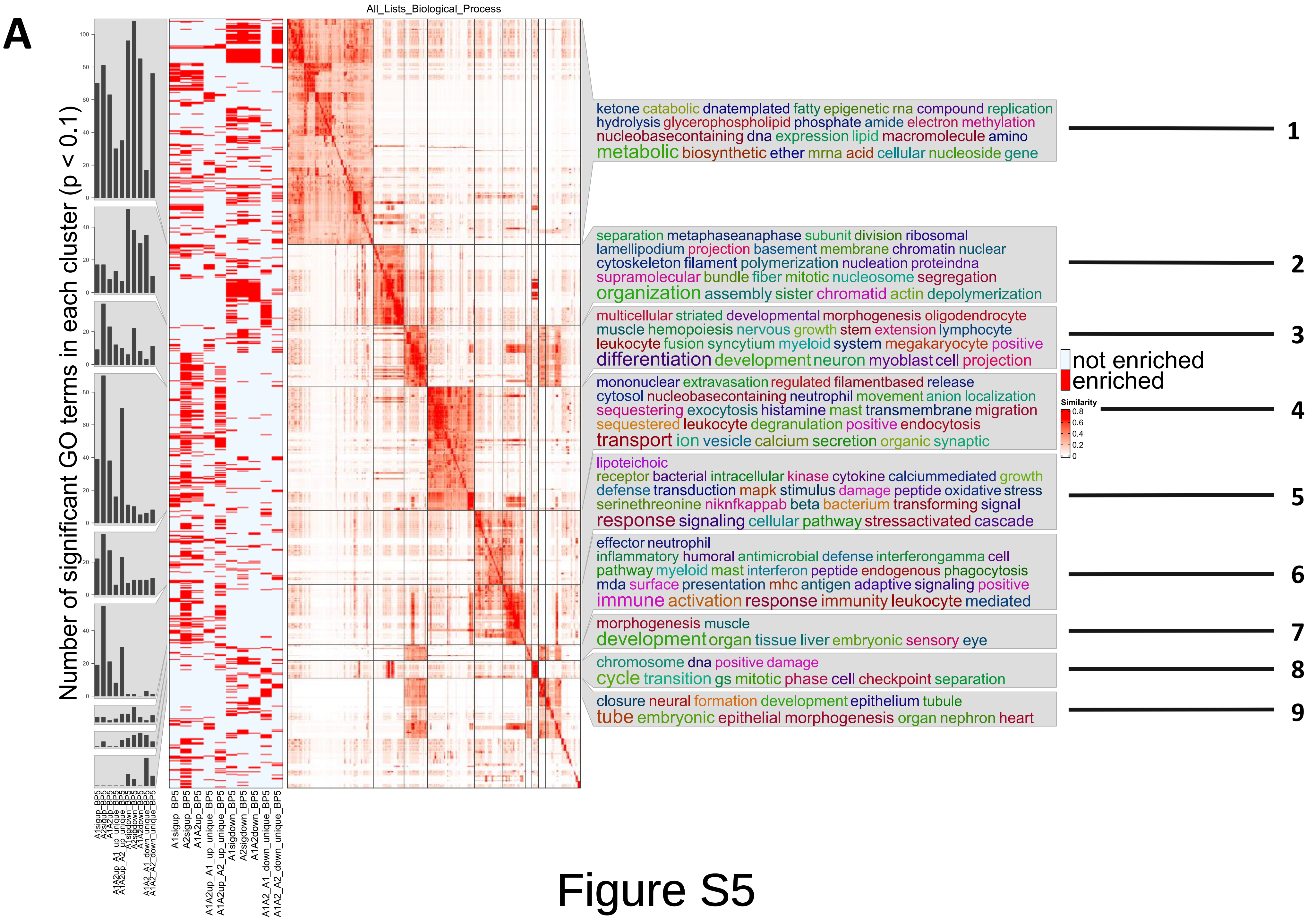
A
All_Lists_Biological_Process
Number of significant GO terms in each cluster (p < 0.1)
ketone catabolic dnatemplated fatty epigenetic rna compound replication hydrolysis glycerophospholipid phosphate amide electron methylation nucleobasecontaining dna expression lipid macromolecule amino metabolic biosynthetic ether mrna acid cellular nucleoside gene
separation metaphaseanaphase subunit division ribosomal lamellipodium projection basement membrane chromatin nuclear cytoskeleton filament polymerization nucleation proteindna supramolecular bundle fiber mitotic nucleosome segregation organization assembly sister chromatid actin depolymerization
multicellular striated developmental morphogenesis oligodendrocyte muscle hemopoiesis nervous growth stem extension lymphocyte leukocyte fusion syncytium myeloid system megakaryocyte positive differentiation development neuron myoblast cell projection
mononuclear extravasation regulated filamentbased release cytosol nucleobasecontaining neutrophil movement anion localization sequestering exocytosis histamine mast transmembrane migration sequestered leukocyte degranulation positive endocytosis transport ion vesicle calcium secretion organic synaptic
lipoteichoic receptor bacterial intracellular kinase cytokine calciummediated growth defense transduction mapk stimulus damage peptide oxidative stress serinethreonine niknfkappab beta bacterium transforming signal response signaling cellular pathway stressactivated cascade
effector neutrophil inflammatory humoral antimicrobial defense interferongamma cell pathway myeloid mast interferon peptide endogenous phagocytosis mda surface presentation mhc antigen adaptive signaling positive immune activation response immunity leukocyte mediated
morphogenesis muscle development organ tissue liver embryonic sensory eye
chromosome dna positive damage cycle transition gs mitotic phase cell checkpoint separation
closure neural formation development epithelium tubule tube embryonic epithelial morphogenesis organ nephron heart
not enriched
enriched
Similarity
0.8
0.6
0.4
0.2
0
1
2
3
4
5
6
7
8
9
A1sigup_BP5
A2sigup_BP5
A1A2up_BP5
A1A2up_A1_up_unique_BP5
A1A2up_A2_up_unique_BP5
A1sigdown_BP5
A2sigdown_BP5
A1A2down_BP5
A1A2_A1_down_unique_BP5
A1A2_A2_down_unique_BP5

Figure S5

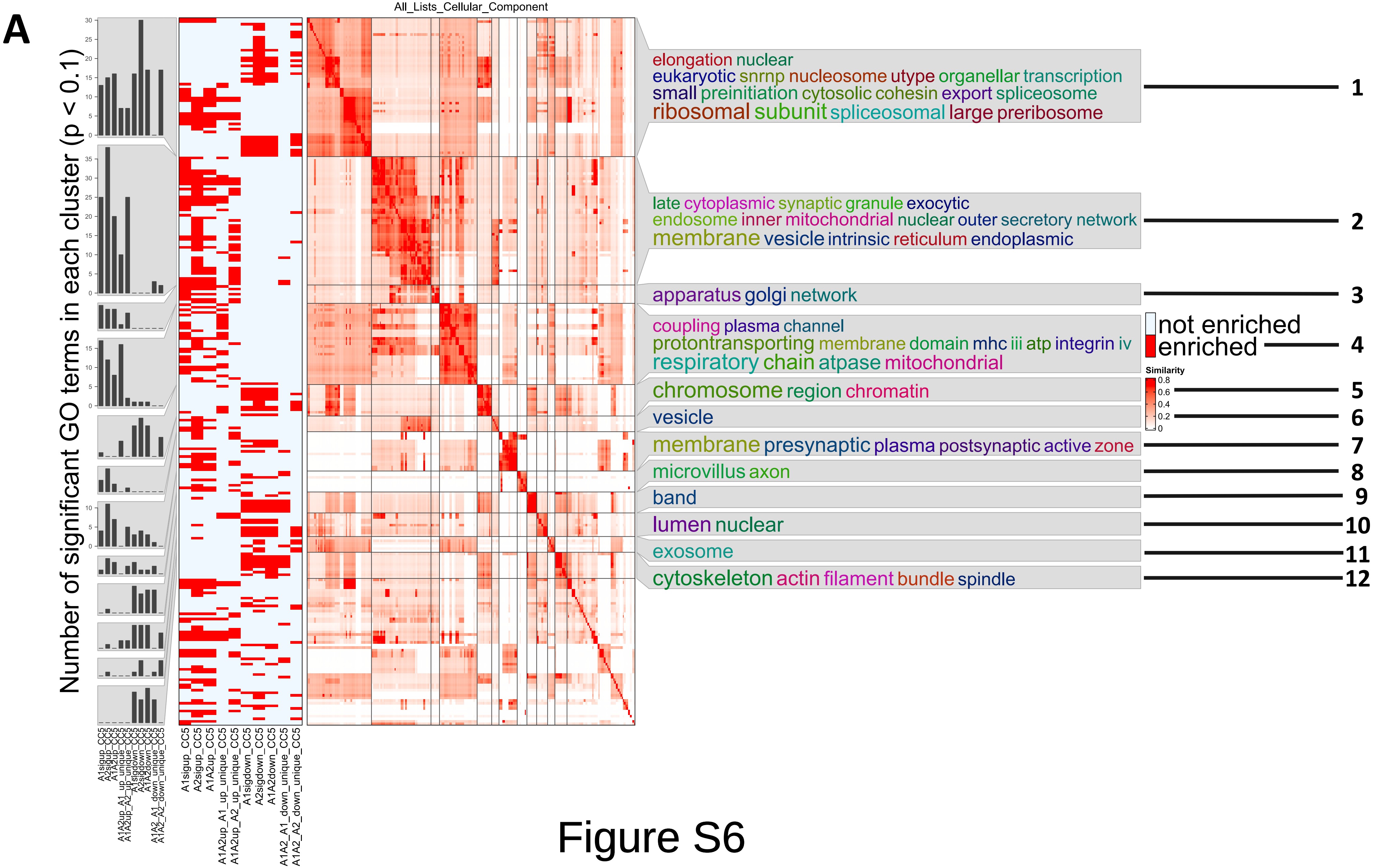
A
All_Lists_Cellular_Component
Number of significant GO terms in each cluster (p < 0.1)
A1sigup_CC5
A2sigup_CC5
A1A2up_CC5
A1A2up_A1_up_unique_CC5
A1A2up_A2_up_unique_CC5
A1sigdown_CC5
A2sigdown_CC5
A1A2down_CC5
A1A2_A1_down_unique_CC5
A1A2_A2_down_unique_CC5
elongation nuclear eukaryotic snrnp nucleosome utype organellar transcription small preinitiation cytosolic cohesin export spliceosome ribosomal subunit spliceosomal large preribosome
1
late cytoplasmic synaptic granule exocytic endosome inner mitochondrial nuclear outer secretory network membrane vesicle intrinsic reticulum endoplasmic
2
apparatus golgi network
3
coupling plasma channel protontransporting membrane domain mhc iii atp integrin iv respiratory chain atpase mitochondrial
4
chromosome region chromatin
5
vesicle
6
membrane presynaptic plasma postsynaptic active zone
7
microvillus axon
8
band
9
lumen nuclear
10
exosome
11
cytoskeleton actin filament bundle spindle
12
not enriched
enriched
Similarity
0.8
0.6
0.4
0.2
0

Figure S6

Figure S7

A

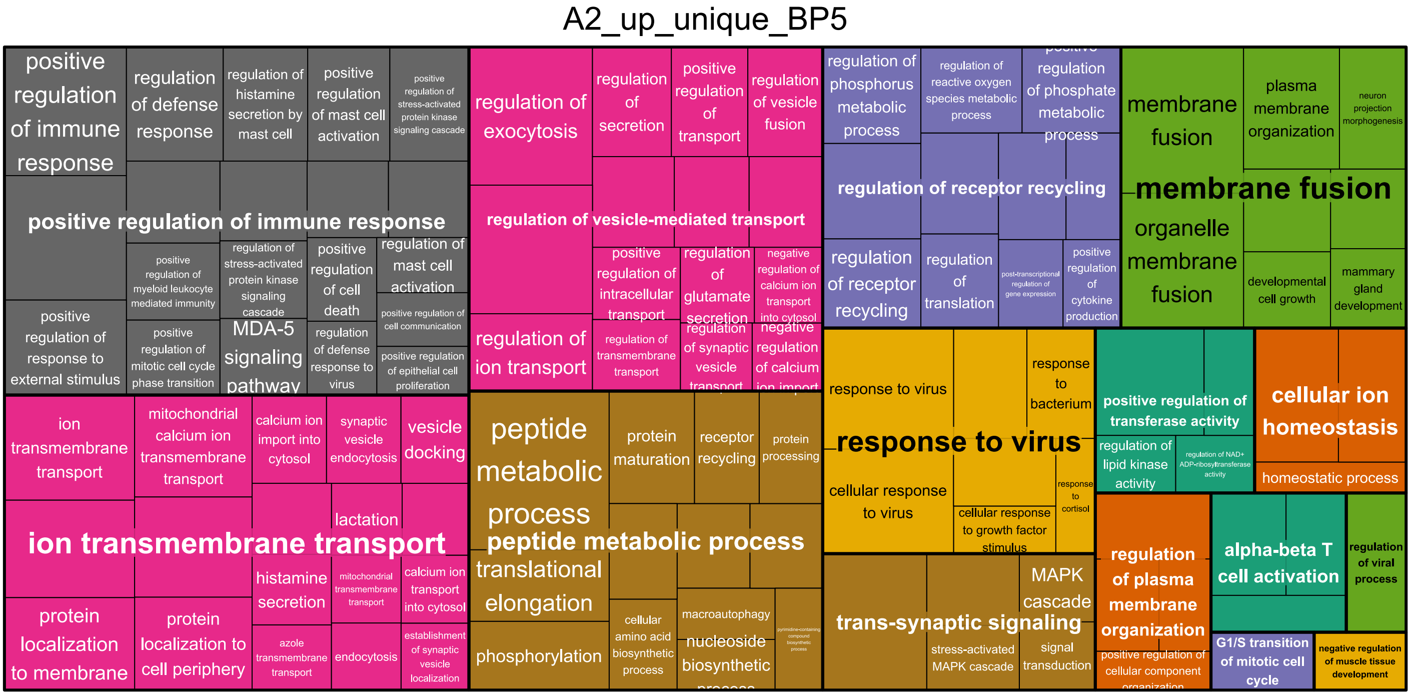

A2_up_unique_BP5
positive regulation of immune response
regulation of defense response
regulation of histamine secretion by mast cell
positive regulation of mast cell activation
positive regulation of stress-activated protein kinase signaling cascade
positive regulation of myeloid leukocyte mediated immunity
regulation of stress-activated protein kinase signaling cascade
positive regulation of cell death
regulation of mast cell activation
positive regulation of response to external stimulus
positive regulation of mitotic cell cycle phase transition
MDA-5 signaling pathway
regulation of defense response to virus
positive regulation of cell communication
positive regulation of epithelial cell proliferation
regulation of exocytosis
regulation of secretion
positive regulation of transport
regulation of vesicle fusion
regulation of vesicle-mediated transport
positive regulation of intracellular transport
regulation of glutamate secretion
negative regulation of calcium ion transport into cytosol
regulation of ion transport
regulation of transmembrane transport
regulation of synaptic vesicle transport
negative regulation of calcium ion import
regulation of phosphorus metabolic process
regulation of reactive oxygen species metabolic process
positive regulation of phosphate metabolic process
regulation of receptor recycling
regulation of translation
post-transcriptional regulation of gene expression
positive regulation of cytokine production
membrane fusion
plasma membrane organization
neuron projection morphogenesis
organelle membrane fusion
developmental cell growth
mammary gland development
ion transmembrane transport
mitochondrial calcium ion transmembrane transport
calcium ion import into cytosol
synaptic vesicle endocytosis
vesicle docking
lactation
histamine secretion
mitochondrial transmembrane transport
calcium ion transport into cytosol
protein localization to membrane
protein localization to cell periphery
azole transmembrane transport
endocytosis
establishment of synaptic vesicle localization
peptide metabolic process
protein maturation
receptor recycling
protein processing
translational elongation
phosphorylation
cellular amino acid biosynthetic process
macroautophagy
nucleoside biosynthetic process
pyrimidine-containing compound biosynthetic process
response to virus
response to bacterium
cellular response to virus
cellular response to growth factor stimulus
response to cortisol
trans-synaptic signaling
stress-activated MAPK cascade
MAPK cascade
signal transduction
positive regulation of transferase activity
regulation of lipid kinase activity
regulation of NAD+ ADP-ribosyltransferase activity
cellular ion homeostasis
homeostatic process
regulation of plasma membrane organization
positive regulation of cellular component organization
alpha-beta T cell activation
regulation of viral process
G1/S transition of mitotic cell cycle
negative regulation of muscle tissue development

B

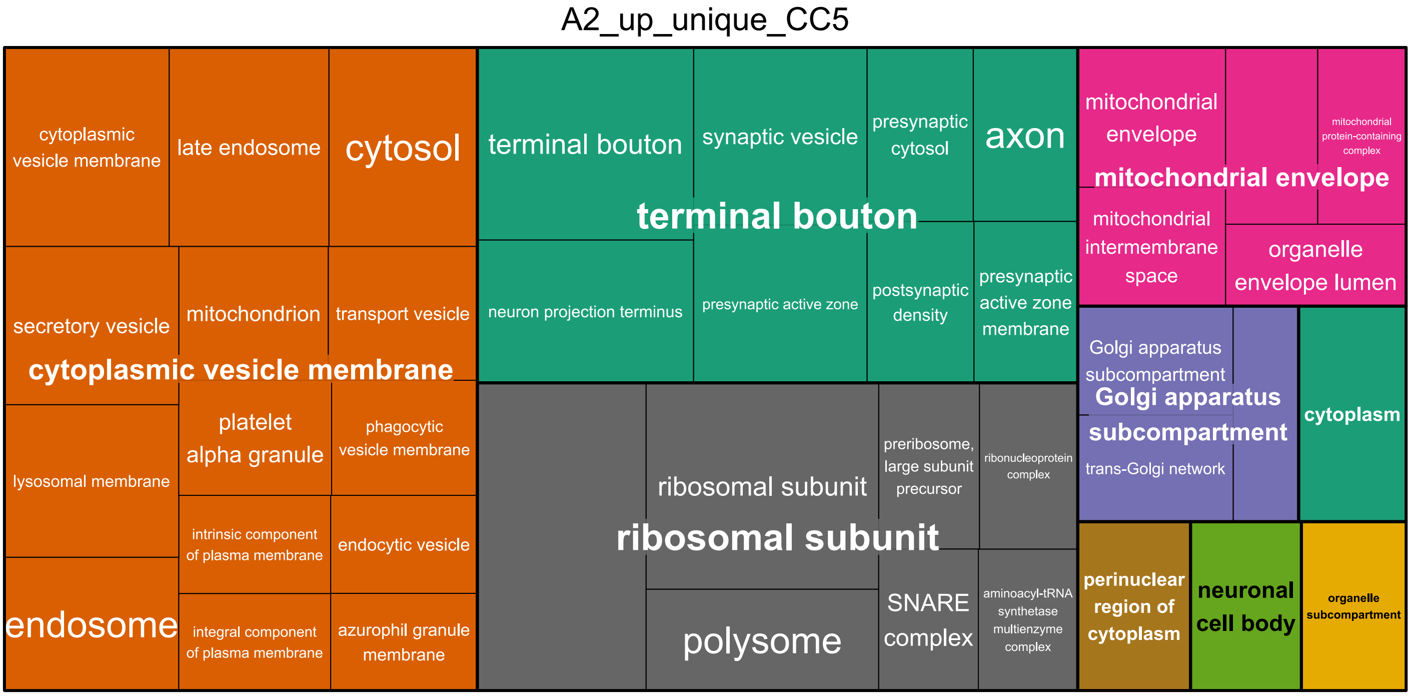

A2_up_unique_CC5
cytoplasmic vesicle membrane
late endosome
cytosol
secretory vesicle
mitochondrion
transport vesicle
lysosomal membrane
platelet alpha granule
phagocytic vesicle membrane
intrinsic component of plasma membrane
endocytic vesicle
endosome
integral component of plasma membrane
azurophil granule membrane
terminal bouton
synaptic vesicle
presynaptic cytosol
axon
neuron projection terminus
presynaptic active zone
postsynaptic density
presynaptic active zone membrane
mitochondrial envelope
mitochondrial protein-containing complex
mitochondrial intermembrane space
organelle envelope lumen
Golgi apparatus subcompartment
trans-Golgi network
cytoplasm
ribosomal subunit
preribosome, large subunit precursor
ribonucleoprotein complex
polysome
SNARE complex
aminoacyl-tRNA synthetase multienzyme complex
perinuclear region of cytoplasm
neuronal cell body
organelle subcompartment

Figure S8

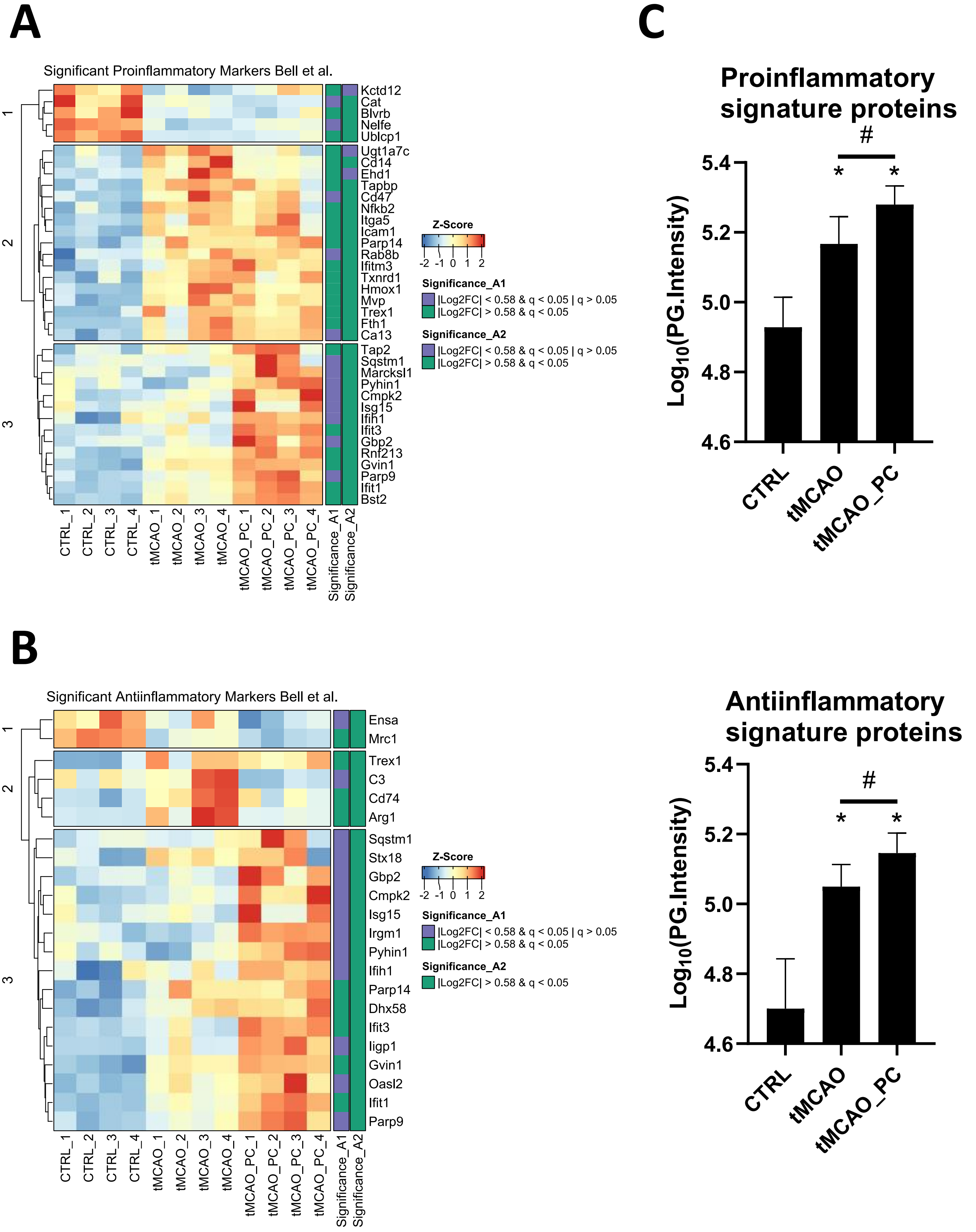
A
Significant Proinflammatory Markers Bell et al.
1
2
3
Kctd12
Cat
Blvrb
Nelfe
Ublcp1
Ugt1a7c
Cd14
Ehd1
Tapbp
Cd47
Nfkb2
Itga5
Icam1
Parp14
Rab8b
Ifitm3
Txnrd1
Hmox1
Mvp
Trex1
Fth1
Ca13
Tap2
Sqstm1
Marcksl1
Pyhin1
Cmpk2
Isg15
Ifih1
Ifit3
Gbp2
Rnf213
Gvin1
Parp9
Ifit1
Bst2
CTRL_1
CTRL_2
CTRL_3
CTRL_4
tMCAO_1
tMCAO_2
tMCAO_3
tMCAO_4
tMCAO_PC_1
tMCAO_PC_2
tMCAO_PC_3
tMCAO_PC_4
Significance_A1
Significance_A2
Z-Score
-2 -1 0 1 2
Significance_A1
|Log2FC| < 0.58 & q < 0.05 | q > 0.05
|Log2FC| > 0.58 & q < 0.05
Significance_A2
|Log2FC| < 0.58 & q < 0.05 | q > 0.05
|Log2FC| > 0.58 & q < 0.05
B
Significant Antiinflammatory Markers Bell et al.
1
2
3
Ensa
Mrc1
Trex1
C3
Cd74
Arg1
Sqstm1
Stx18
Gbp2
Cmpk2
Isg15
Irgm1
Pyhin1
Ifih1
Parp14
Dhx58
Ifit3
Iigp1
Gvin1
Oasl2
Ifit1
Parp9
CTRL_1
CTRL_2
CTRL_3
CTRL_4
tMCAO_1
tMCAO_2
tMCAO_3
tMCAO_4
tMCAO_PC_1
tMCAO_PC_2
tMCAO_PC_3
tMCAO_PC_4
Significance_A1
Significance_A2
Z-Score
-2 -1 0 1 2
Significance_A1
|Log2FC| < 0.58 & q < 0.05 | q > 0.05
|Log2FC| > 0.58 & q < 0.05
Significance_A2
|Log2FC| > 0.58 & q < 0.05
C
Proinflammatory signature proteins
Log10(PG.Intensity)
5.4
5.2
5.0
4.8
4.6
#
*
*
CTRL
tMCAO
tMCAO_PC
Antiinflammatory signature proteins
Log10(PG.Intensity)
5.4
5.2
5.0
4.8
4.6
#
*
*
CTRL
tMCAO
tMCAO_PC

Figure S9

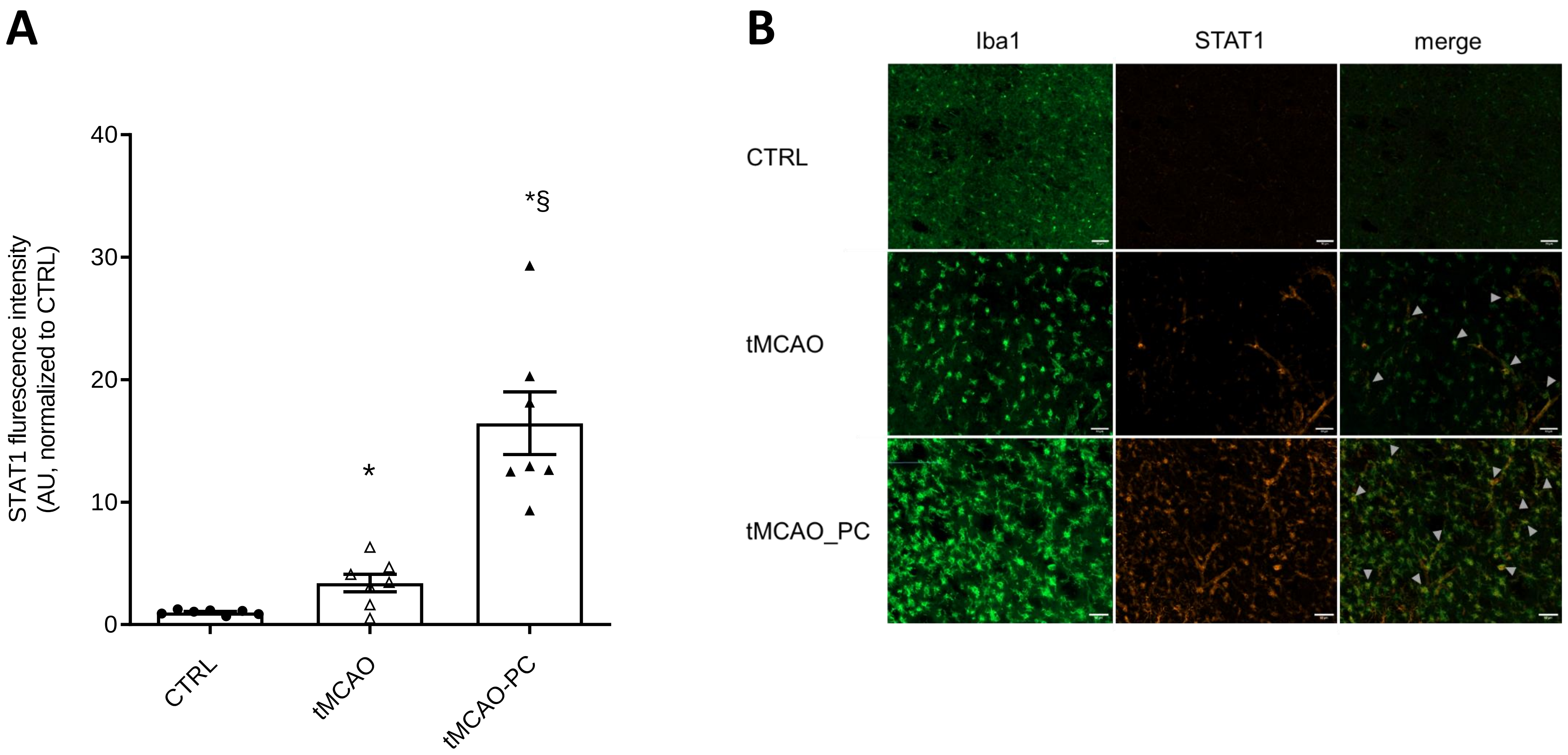

A
STAT1 flurescence intensity (AU, normalized to CTRL)
40
30
20
10
0
*§
*
CTRL
tMCAO
tMCAO-PC
B
Iba1
STAT1
merge
CTRL
tMCAO
tMCAO_PC
