# Supplementary material for: Impact of inflammatory preconditioning on murine microglial proteome response induced by focal ischemic brain injury
Source: Front Immunol. 2024 Apr 9;15:1227355. doi: 10.3389/fimmu.2024.1227355 (PMC11036884; doi:10.3389/fimmu.2024.1227355)
Supplement: Supplementary Figure 2 — Correlation plot analysis. Depiction of the Log2 fold changes of the ten most up- and down-regulated proteins in the Depiction of the A1 and A2 comparison. [file DataSheet_2.pdf]

Supplementary material:

Dario Lucas Helbing et al.: Impact of inflammatory preconditioning on murine microglial proteome response induced by focal ischemic brain injury

Results

Table 2 Suppl: Effect of LPS preconditioning (0.8 µg/g b.w., i.p.) on clinical course before tMCAO administration [1,2].

Clinical score	2 days before tMCAO	1 day before tMCAO	immediately before tMCAO
tMCAO	1.0 (1.0; 1.0)	1.0 (1.0; 1.0)	1.0 (1.0; 1.0)
tMCAO_PC	1.25 (1.0; 1.5) $^{\#}$	1.0 (1.0; 1.5) $^{\#}$	1.0 (1.0; 1.25)

Values are given as medians, with the first and third quartiles in parenthesis; n = 13 per group.

two-way ANOVA and post-hoc Holm-Sidak test, $^{\#}$ $p < 0.05$ vs. tMCAO and tMCAO_PC at given time point.

References

1. Gonnert FA, Recknagel P, Seidel M, Jbeily N, Dahlke K, Bockmeyer CL, Winning J, Losche W, Claus RA, Bauer M: **Characteristics of clinical sepsis reflected in a reliable and reproducible rodent sepsis model.** *J Surg Res* 2011, **170:**e123-134.
2. Lang GP, Ndongson-Dongmo B, Lajqi T, Brodhun M, Han Y, Wetzker R, Frasch MG, Bauer R: **Impact of ambient temperature on inflammation-induced encephalopathy in endotoxemic mice-role of phosphoinositide 3-kinase gamma.** *J Neuroinflammation* 2020, **17:**292.
